# Supplementary material for: Chondrocyte fatty acid oxidation drives osteoarthritis via SOX9 degradation and epigenetic regulation
Source: Nat Commun. 2025 May 27;16:4892. doi: 10.1038/s41467-025-60037-4 (PMC12117060; doi:10.1038/s41467-025-60037-4)
Supplement: Supplementary file 1 — Supplementary Information [file 41467_2025_60037_MOESM1_ESM.pdf]

Chondrocyte fatty acid oxidation drives osteoarthritis via SOX9 degradation and epigenetic regulation

Zixuan Mei,[1,2, #] Kamuran Yilamu,[1,2, #] Weiyu Ni,[1,2, #] Panyang Shen, [1,2, #] Nan Pan, [1,2] Huasen Chen, [1,2] Yingfeng Su, [1,2] Lei Guo,[3] Qunan Sun, [4] Zhaomei Li,[5] Dongdong Huang,[3] Xiangqian Fang, [1,2] Shunwu Fan,[1,2, *] Haitao Zhang,[1,2, *] and Shuying Shen[1,2, *]

Inventory of Supporting Information

Figure S1. Lipid accumulation in cartilage accelerates OA progression.
Figure S2. Chondrocytes uptake fatty acids from synovial fluid, contributing to cartilage lipid accumulation.
Figure S3. Acetyl-CoA orchestrates protein acetylation dynamics, rewiring FAO in chondrocytes.
Figure S4. HADHA K728ac is up-regulated under lipid stress and boosts FAO in chondrocytes.
Figure S5. Inhibition of FAO attenuates OA progression *in vitro*.
Figure S6. Inhibition of FAO attenuates OA progression *in vivo*.
Figure S7. FAO inhibited AMPK activity and increased SOX9 degradation.
Figure S8. Phosphorylation of SOX9 impedes its interaction with TRIM9 and enhances its stability.
Figure S9. FFA epigenetically regulates cartilage ECM metabolism.
Figure S10. Isolation of RBC-exosomes (RBC-Exo) and production of CAP-RBC-Exo/TMZ.
Supplementary Table 1. The medical records of patients underwent knee replacement surgery.
Supplementary Table 2. The medical records and laboratory findings from patients underwent synovial fluid collection.
Supplementary Table 3. Oligonucleotide and primer list.
Supplementary Table 4. The OA cartilage histopathology grade.
Supplementary Table 5. The recommended semi-quantitative scoring system (OARSI score).
Supplementary Table 6. The Mankin Histology histopathology grading system.
Supplementary Table 7. Multiple reaction monitoring parameters.
Supplementary Table 8. Key resources table.
Supplementary Data 1. Lipidomic data, related to Figures S1i and S1j.
Supplementary Data 2. Proteomics data, related to Figures S3g and S3h.
Supplementary Data 3. Acetylome data, related to Figures 2c,2d and S3j.
Supplementary Data 4. CUT&Tag-sequencing data, related to Figures 5c,5d,5e and S9b.
Source Data.

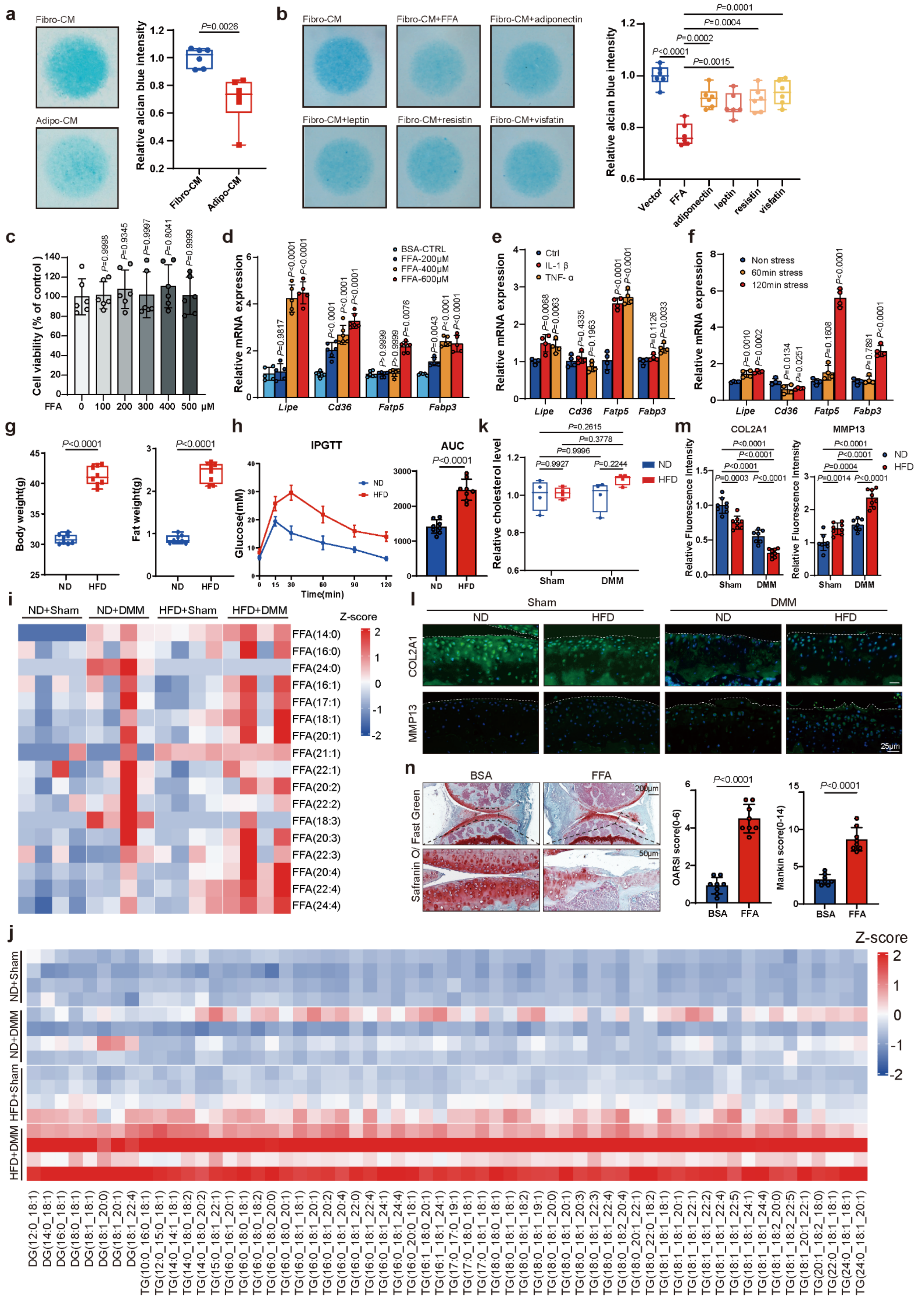

Figure S1. Lipid accumulation in cartilage accelerates OA progression. (a-b) Micromass culture and alcian

blue staining of primary mouse chondrocytes treated as indicated (n = 6). (c) Cell viability of mouse chondrocytes treated with specific concentrations of FFA (n=6). (d) Changes in *Lipe*, *Cd36*, *Fatp5*, and *Fabp3* mRNA levels in primary murine chondrocytes, treated with FFA, as determined via RT-qPCR analysis (n= 5 for *Lipe and Fabp3*; n= 6 for *Cd36* and *Fatp5*). (e) Changes in *Lipe*, *Cd36*, *Fatp5*, and *Fabp3* mRNA levels in primary murine chondrocytes, treated with IL-1β and TNF-α, as determined via RT-qPCR analysis (n = 4). (f) Changes in *Lipe*, *Cd36*, *Fatp5*, and *Fabp3* mRNA levels in primary murine chondrocytes treated with tensile-stress loading, as determined via RT-qPCR analysis (n = 4). (g) Body weight and inguinal adipose tissue weight of mice indicated in Figure 1f before sacrifice (n = 8). (h) Intraperitoneal glucose tolerance test (IPGTT) results before sacrifice (n = 8). (i) Heatmap showing the levels of FFAs with different lengths in cartilage samples from mice indicated in Figure 1f. (j) Heatmap showing the top 60 upregulated GL species containing FFAs with different lengths in cartilage samples from mice as indicated in Figure 1f. (k) Relative cholesterol level in cartilage samples from mice indicated in Figure 1f (n = 4). (l-m) IF staining for COL2A1 and MMP13 in mouse knee joints indicated in Figure 1i (n = 7 for ND DMM group; n = 8 for ND Sham, HFD Sham, and HFD DMM groups). (n) Safranin O/Fast green staining of knee joints from mice treated with FFA or BSA arthrocentesis (n = 8). (a, b, g, and k) Data are minimum to maximum: each point represents an individual biological replication, box from 25th to 75th percentiles; center line at median; tukey whiskers, as determined via unpaired two-tailed *t*-test (a, b, and g) or two-way ANOVA followed by tukey's multiple-comparisons test (k). (c-f, h, and m-n) Data are mean ±SD, as determined by one-way ANOVA followed by Dunn's multiple-comparisons test (c, f) or two-tailed *t*-test or nonparametric two-tailed Mann–Whitney test (e, h, and m-n). Source data are provided as a Source Data file.

a

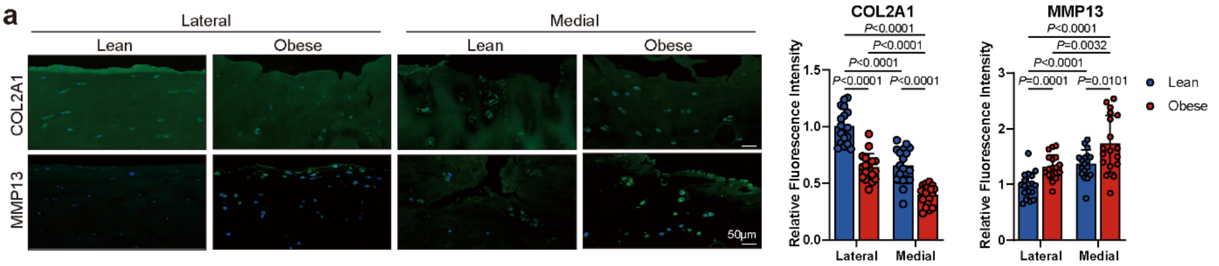

b

Clinic characteristics of the subjects in each set

	Mild(n=40)	Middle(n=36)	Severe(n=44)	*P* value	Lean(n=52)	Obese(n=40)	*P* value
Age(years)	58.60±11.44	64.06±7.78	68.07±8.19	<0.0001	62.88±11.36	61.50±9.22	0.5318
Gender(male/female)	10M/30F	8M/28F	16M/28F	0.3201	17M/35F	12M/28F	0.7829
Height(cm)	161.6±8.09	161.0±7.69	163.2±8.17	0.4239	162±8.50	162.3±8.23	0.8704
Body weight(kg)	64.5±13.04	68.24±11.80	69.88±12.71	0.1414	57.31±7.84	80.19±8.81	<0.0001
BMI	24.71±4.86	26.25±3.45	26.29±4.11	0.1615	21.78±1.86	30.57±2.03	<0.0001

c

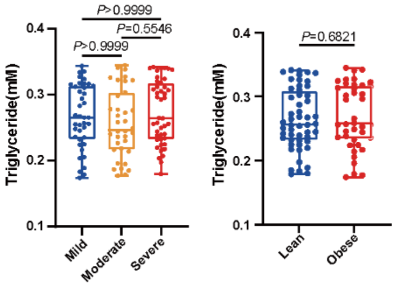

d

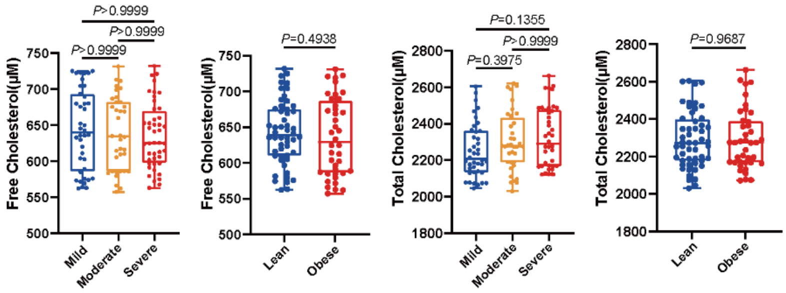

Figure S2. Chondrocytes uptake fatty acids from synovial fluid, contributing to cartilage lipid accumulation. (a) IF staining for COL2A1 and MMP13 in human knee cartilage tissues indicated in Figure 1j (n = 18). (b) Clinical data of patients, including age, gender, height, body weight, and BMI. (c) The level of total triglycerides in synovial fluids from patients with mild (n = 40), moderate (n = 36), and severe (n = 44) OA (Left), and from patients classified as Lean (n = 52) or with obesity (n = 40) (Right). (d) The levels of free cholesterols and total cholesterols in synovial fluids from patients with mild (n = 40), moderate (n = 36), and severe (n = 44) OA (Left), and from patients classified as Lean (n = 52) or with obesity (n = 40) (Right). (a) Data are mean ±SD, as determined by two-tailed *t*-test or nonparametric two-tailed Mann–Whitney test. (c-d) Data are minimum to maximum: each point represents an individual biological replication, box from 25th to 75th percentiles; center line at median; tukey whiskers, as determined via nonparametric Kruskal–Wallis test followed by Dunn's multiple comparisons test, or nonparametric two-tailed Mann–Whitney test. Source data are provided as a Source Data file.

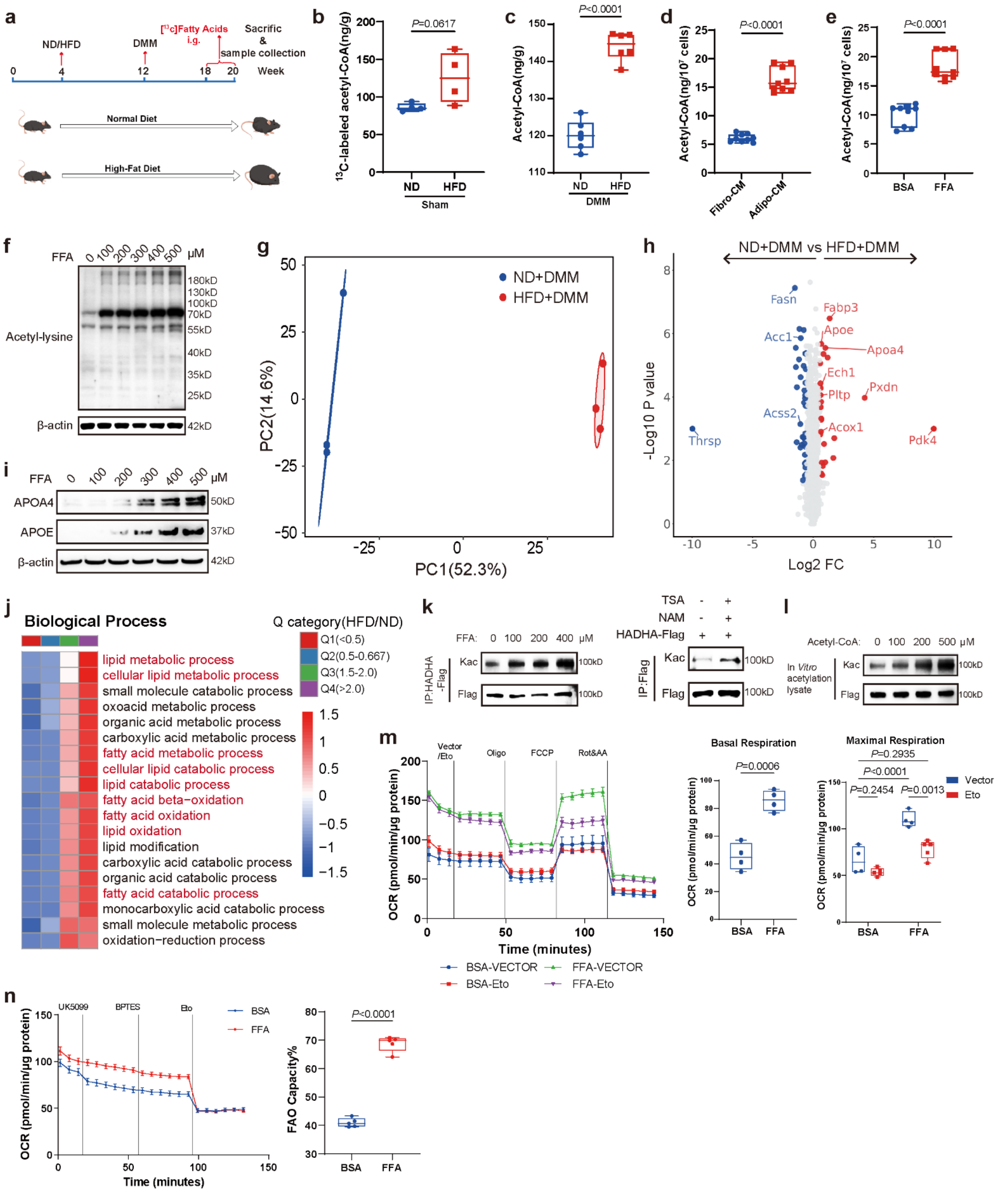

Figure S3. Acetyl-CoA orchestrates protein acetylation dynamics, rewiring FAO in chondrocytes. (a) Illustration of diet change and destabilization of the DMM surgery or sham operation in male C57BL/6J mice, two weeks before sacrifice; all mice were orally gavaged with [^{13}C] fatty acids every day, by figdraw.com. (b) Quantification of ^{13}C-labeled acetyl-CoA levels in cartilage samples from mice that underwent Sham operation, as indicated above (n = 4). Data represent the minimum to maximum; each point represents an individual biological replicate, as determined via nonparametric two-tailed Mann–Whitney test. (c-e) The level of acetyl-CoA in cartilage samples from ND+DMM and HFD+DMM mice (c, n = 6) and in primary murine chondrocytes treated with adipo-CM or fibro-CM (d, n = 9), FFA, or BSA (e, n = 9), assessed via ELISA. Data represent the minimum to maximum; each point represents an individual biological replicate, as determined via unpaired two-tailed *t*-test. (f) Western

blot analysis of acetyl-lysine from whole cell lysate harvested from primary murine chondrocytes treated with FFA. (g) PCA analysis for visualizing the protein alterations among experimental groups. (h) Volcano plot analysis of proteins altered in HFD+DMM versus ND+DMM groups, via two-tailed t-test. (i) Immunoblot detection of APOA4 and APOE in primary mouse chondrocytes treated with FFA. (j) GO enrichment analysis of acetylation sites showing enriched BP terms in HFD+DMM versus ND+DMM groups ($P < 0.05$), via two-tailed Fisher's exact test. (k-l) Immunoblots showing HADHA acetylation when overexpressed in C28/I2 cells treated as indicated or *in vitro* acetylation in lysates containing indicated concentrations of acetyl-CoA. (m) Extracellular-flux analysis (EFA) of OCRs. Basal respiration and maximal respiration were calculated using Wave 2.4.0 (n = 4 for Vector treated groups; n = 5 for Eto treated groups). Data are mean ±SEM or minimum to maximum: each point represents an individual biological replicate, as determined via unpaired two-tailed t-test or two-way ANOVA followed by Tukey's multiple-comparisons test. (n) Mitochondrial fuel oxidation analysis (n = 5). Data are mean ±SEM or minimum to maximum: each point represents an individual biological replicate, as determined via unpaired two-tailed t-test. The western blot data are representative of three independent experiments. Source data are provided as a Source Data file.

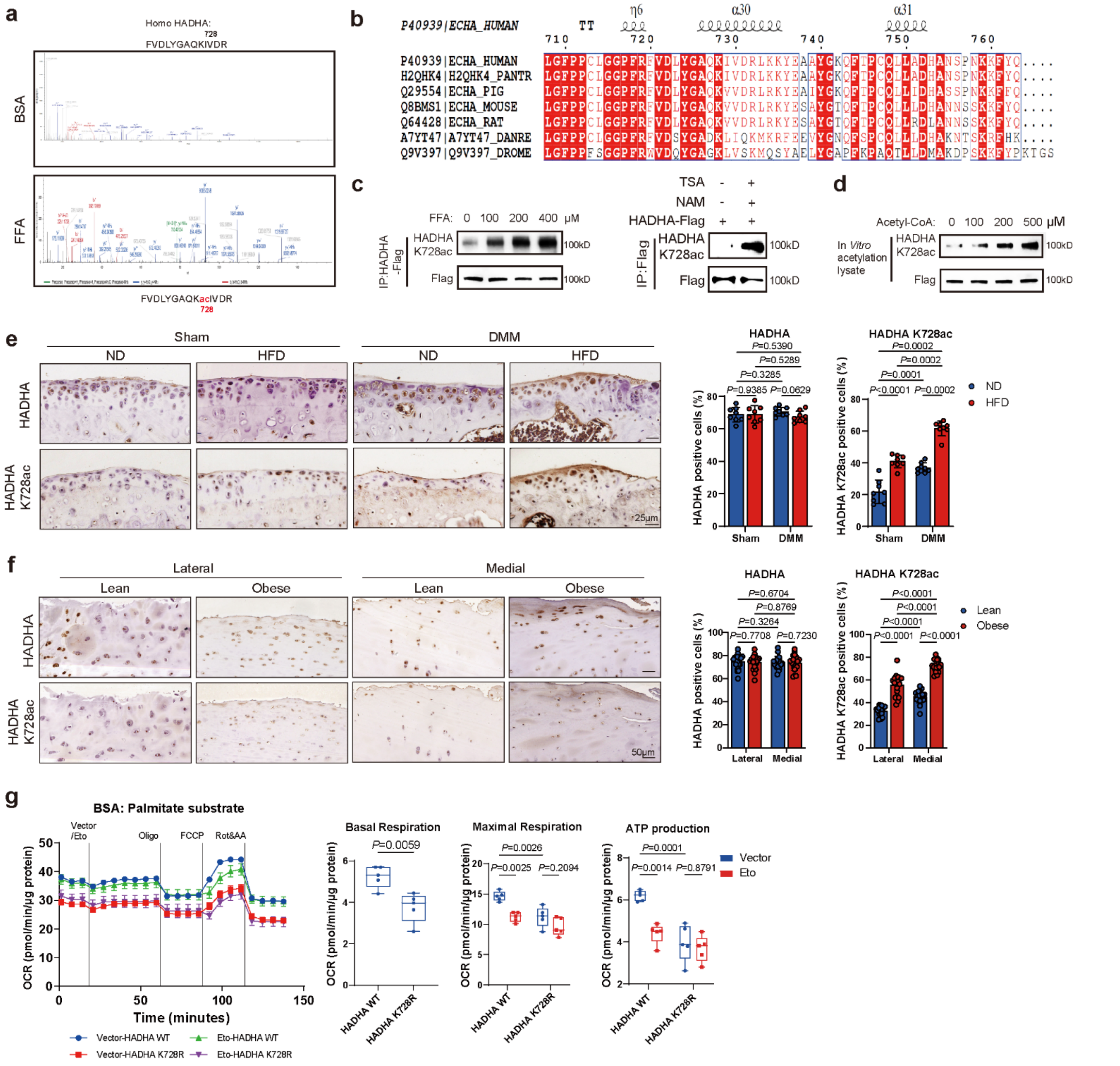

Figure S4. HADHA K728ac is up-regulated under lipid stress and boosts FAO in chondrocytes. (a) UPLC-MS/MS for identifying the acetylation site on HADHA in C28/I2 cells treated with FFA or BSA. (b) Structure-based sequence alignments of HADHA amino acids 708 to 763, carried out using ESPript 3.0. (c-d) Immunoblot showing HADHA K728 acetylation when overexpressed in C28/I2 cells treated as indicated or *in vitro* acetylation in lysates containing indicated concentrations of acetyl-CoA. (e) IHC staining for HADHA and HADHA K728ac in mouse knee joints indicated in Figure 1h (n = 8). Data represent the mean± SD, as determined via two-tailed *t*-test or nonparametric two-tailed Mann–Whitney test. (f) IHC staining for HADHA and HADHA K728ac in human knee cartilage indicated in Figure 1j (n = 18 for Lean group; n = 20 for Obese group). Data represent the mean± SD, as determined via two-tailed *t*-test or nonparametric two-tailed Mann–Whitney test. (g) EFA of FAO substrate OCRs. After 24 h of starvation, HADHA-KO C28/I2 cells overexpressing wild-type HADHA or HADHA K728R were treated with BSA-conjugated palmitate 15 min before loading. Basal respiration, maximal respiration, and ATP production were calculated using Wave 2.4.0 (n = 5). Data are mean ±SEM or minimum to maximum: each point represents an individual biological replicate, as determined via unpaired two-tailed t-test or two-way ANOVA followed by Tukey's multiple-comparisons test. The western blot data are representative of three independent experiments. Source data are provided as a Source Data file.

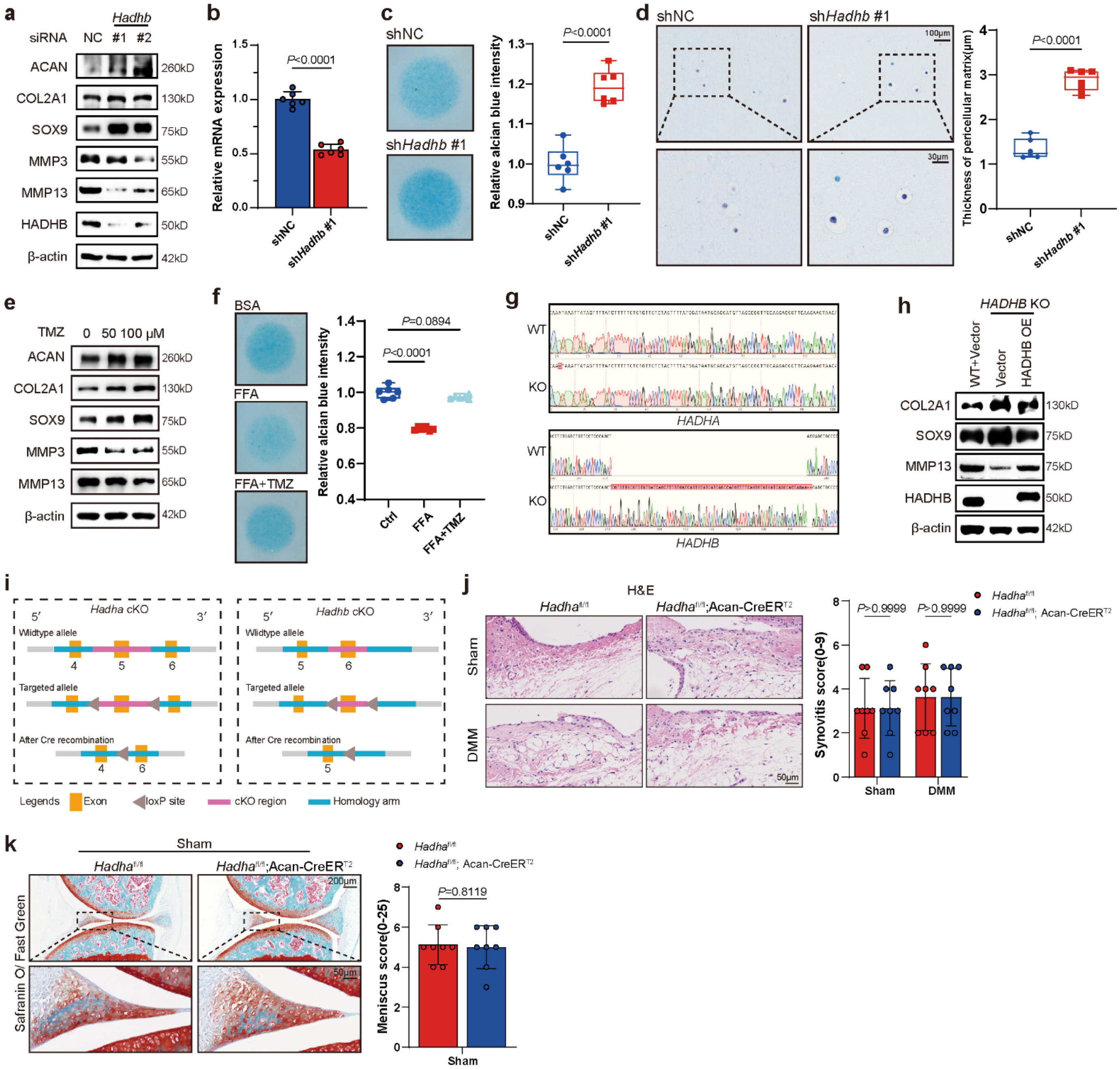

Figure S5. Inhibition of FAO attenuates OA progression *in vitro*. (a) Immunoblot detection of ACAN, COL2A1, SOX9, MMP3, MMP13, and HADHB in mouse chondrocytes transfected with *Hadhb* siRNAs. All cells were treated with 200 μM FFA. (b) mRNA expression levels of *Hadha* in mouse chondrocytes infected with a lentivirus encoding *Hadhb* short hairpin RNA #1 (n = 6). (c-d) Micromass culture(c) or 3D-agarose culture (d), along with alcian blue staining of murine chondrocytes infected with a lentivirus encoding *Hadhb* short hairpin RNA #1 (n = 6). Notably, the images and statistical data of shNC were incorporated into Figure 3c-3d as the experiments were performed simultaneously with a single control experiment. (e) Immunoblot detection of ACAN, COL2A1, SOX9, MMP3, and MMP13 in TMZ-treated mouse chondrocytes. All cells were treated with 200 μM FFA. (f) Micromass culture and alcian blue staining of murine chondrocytes treated with FFA ± TMZ (n = 6). (g) Illustrates the DNA sequence surrounding the sgRNA targeting regions in *HADHA*-KO and *HADHB*-KO C28/I2 cells. (h) Immunoblot detection of COL2A1, SOX9, MMP13, and HADHB in *HADHB*-KO C28/I2 cells overexpressing vector or HADHB. All cells were treated with 200 μM FFA. (i) Gene editing strategy for *Hadha* and *Hadhb* conditional knock-out mouse. (j) Hematoxylin and eosin staining (H&E) of synovial tissues from knee joints of $Hadha^{fl/fl}$ and $Hadha^{fl/fl}$; Acan-creER^{T2} mice. Synovitis scores was used to assess histological changes in the synovial tissues (n = 8). (k) Safranin O/Fast green staining for HADHA in knee joints of $Hadha^{fl/fl}$ and $Hadha^{fl/fl}$; Acan-creER^{T2} mice that underwent sham surgery. Meniscus scores was used to assess histological changes in the meniscus (n = 8). (b, and j-k) Data are mean ± SD, as determined via unpaired two-tailed *t*-test or nonparametric two-tailed Mann–Whitney test. (c-f) Data are

minimum to maximum: each point represents an individual biological replicate, box from 25th to 75th percentiles; center line at median; tukey whiskers, as determined via unpaired two-tailed t-test (c-d) or one-way ANOVA followed by Tukey's multiple-comparisons test (f). The immunoblotting data are representative of three independent experiments. Source data are provided as a Source Data file.

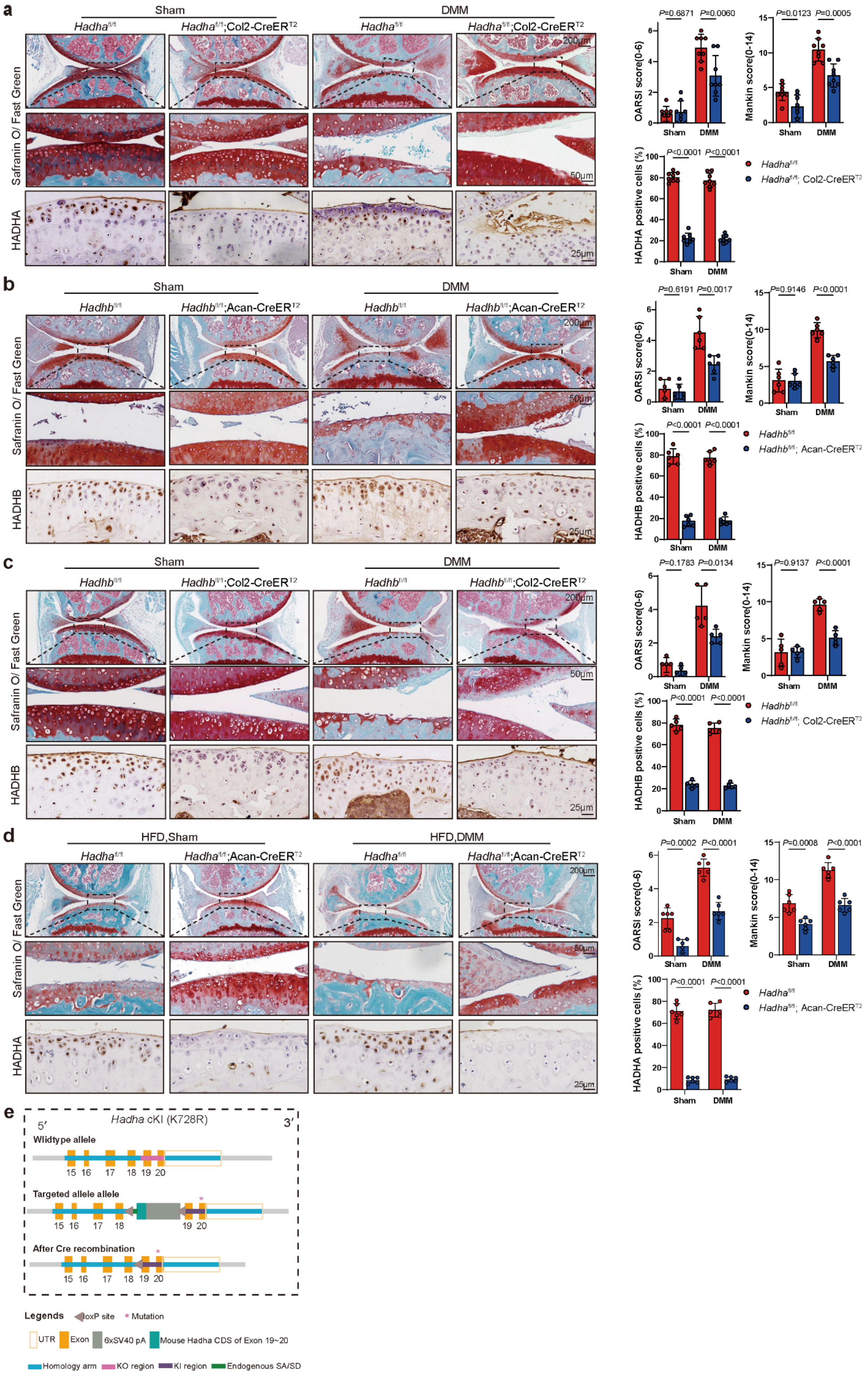

Figure S6. Inhibition of FAO attenuates OA progression *in vivo*. (a) Safranin O/Fast green staining and IHC staining for HADHA in knee joints of *Hadha*$^{fl/fl}$ and *Hadha*$^{fl/fl}$; Col2-creER^{T2} mice that underwent DMM or sham

surgery. Samples were collected 12 weeks after surgery (n = 8). (b) Safranin O/Fast green staining and IHC staining for HADHB in knee joints of *Hadhb*$^{fl/fl}$ and *Hadhb*$^{fl/fl}$; Acan-creER^{T2} mice that underwent DMM or sham surgery. Samples were collected 12 weeks after surgery (n = 6). (c) Safranin O/Fast green staining and IHC staining for HADHB in knee joints of *Hadhb*$^{fl/fl}$ and *Hadhb*$^{fl/fl}$; Col2-creER^{T2} mice that underwent DMM or sham surgery. Samples were collected 12 weeks after surgery (n = 5). (d) Safranin O/Fast green staining and IHC staining for HADHA in knee joints of *Hadha*$^{fl/fl}$ and *Hadha*$^{fl/fl}$; Acan-creER^{T2} mice that underwent DMM or sham surgery at 12 weeks of age. Mice were started on an HFD at 4 weeks, with samples collected 8 weeks after surgery (n = 8). (e) Gene editing strategy for HADHA conditional knock-in (K728R) mouse. Data are mean ± SD, as determined via unpaired two-tailed *t*-test or nonparametric two-tailed Mann–Whitney test. Source data are provided as a Source Data file.

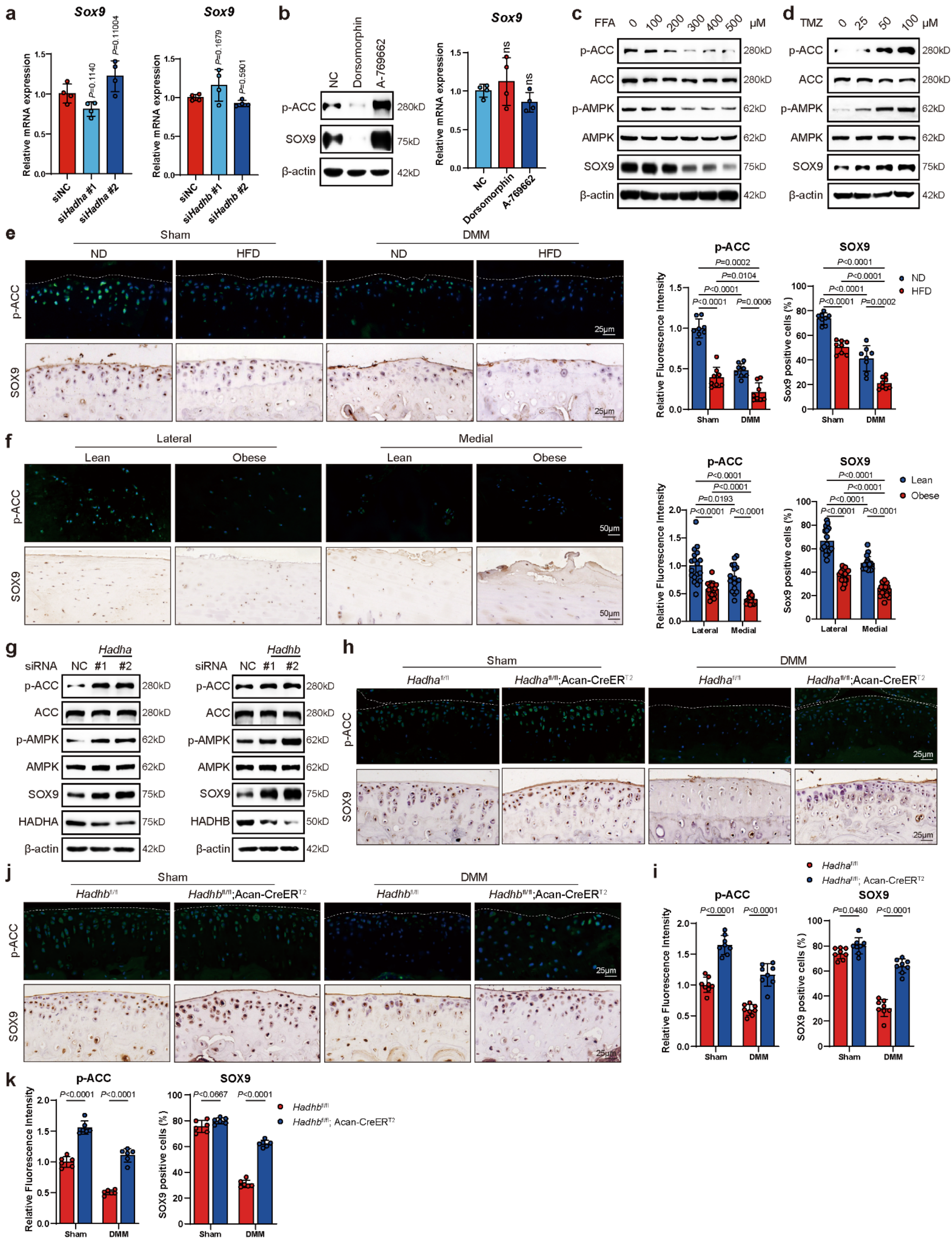

Figure S7. FAO inhibited AMPK activity and increased SOX9 degradation. (a) *Sox9* mRNA levels in mouse chondrocytes transfected with *Hadha* and *Hadhb* siRNAs. (b) Immunoblot of p-ACC and SOX9, and *Sox9* mRNA level in primary mouse chondrocytes treated with dorsomorphin and A-769662 overnight (n = 4). (c-d) Immunoblot of p-ACC/ACC, p-AMPK/AMPK, and SOX9 in primary murine chondrocytes with indicated treatment. (e) IF staining for p-ACC and IHC staining for SOX9 in mouse knee cartilage shown in Figure 1h (n = 8). (f) IF staining for p-ACC

and IHC staining for SOX9 in human knee cartilage shown in Figure 1j (n = 18). (g) Immunoblot of p-ACC/ACC, p-AMPK/AMPK, and SOX9 in primary mouse chondrocytes transfected with *Hadha* and *Hadhb* siRNAs. (h-i) IF staining for p-ACC and IHC staining for SOX9 in mouse knee cartilage indicated in Figure 3h (n = 8). (j-k) IF staining for p-ACC and IHC staining for SOX9 in mouse knee cartilage shown in Figure S6b (n = 6). Data represent the mean ± SD, as determined by one-way ANOVA, test followed by Dunnett's multiple comparisons test (a, b) or unpaired two-tailed t-test or nonparametric two-tailed Mann–Whitney test (e-k). The western blot data are representative of three independent experiments. Source data are provided as a Source Data file.

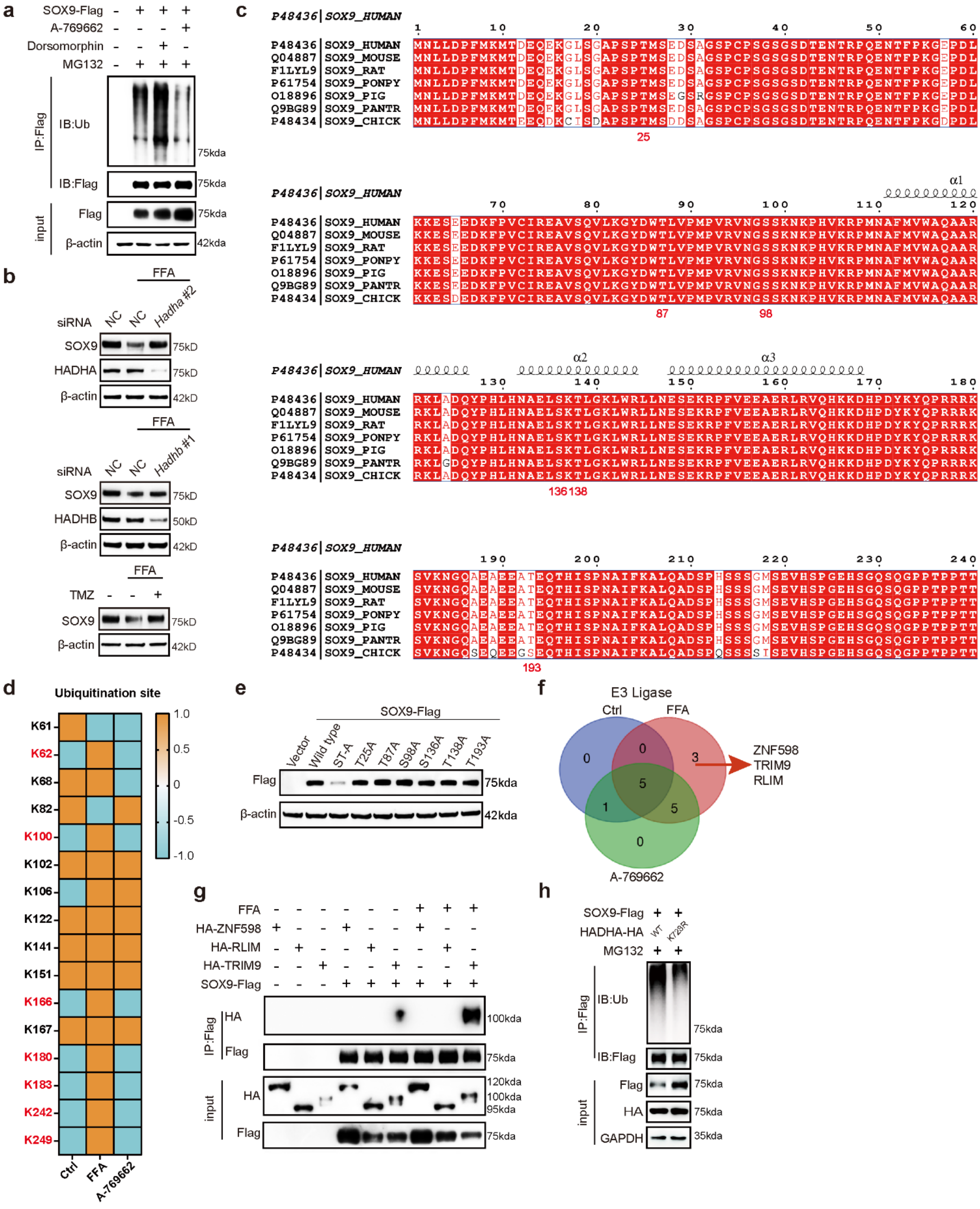

Figure S8. Phosphorylation of SOX9 impedes its interaction with TRIM9 and enhances its stability. (a) Immunoblot detection of ubiquitinated Flag-tagged SOX9 overexpressed in C28/I2 cells via Flag immunoprecipitation. The cells were treated as indicated for 12h and with MG132 for 6 h before harvesting. (b) Immunoblot detection of SOX9 in mouse chondrocytes treated as indicated. (c) Structure-based amino-acid sequence alignments of SOX9 (amino acids 1 to 240 are shown) carried out using ESPript 3.0. (d) Heatmap showing the results of UPLC-MS/MS analysis for identifying ubiquitination sites on the SOX9 protein purified from C28/I2 cells. The presence, absence, or lack of detection of modifications at the specified sites in the SOX9 protein are denoted by ‘1’, ‘–1’, and ‘0’ respectively. (e) Immunoblot analysis of Flag tagged-wild-type or mutated SOX9 in HEK293T cells. The cells were pretreated with CHX for 3 hours. The basal SOX9 protein levels were adjusted to

equivalent amounts by controlling the plasmid transfection dosage. (f) Venn diagram showing that the E3 ubiquitin ligase interacted with SOX9 purified from C28/I2 cells treated as indicated. (g) Co-immunoprecipitation of Flag-tagged SOX9 with HA-tagged ZNF598, RLIM, and TRIM9 in HEK293T cells treated with FFA or BSA. (h) Immunoblot detection of ubiquitinated SOX9, isolated from *HADHA*-KO C28/I2 cells overexpressing wild-type HADHA or HADHA K728R via anti-Flag tag immunoprecipitation. The western blot data are representative of three independent experiments. Source data are provided as a Source Data file.

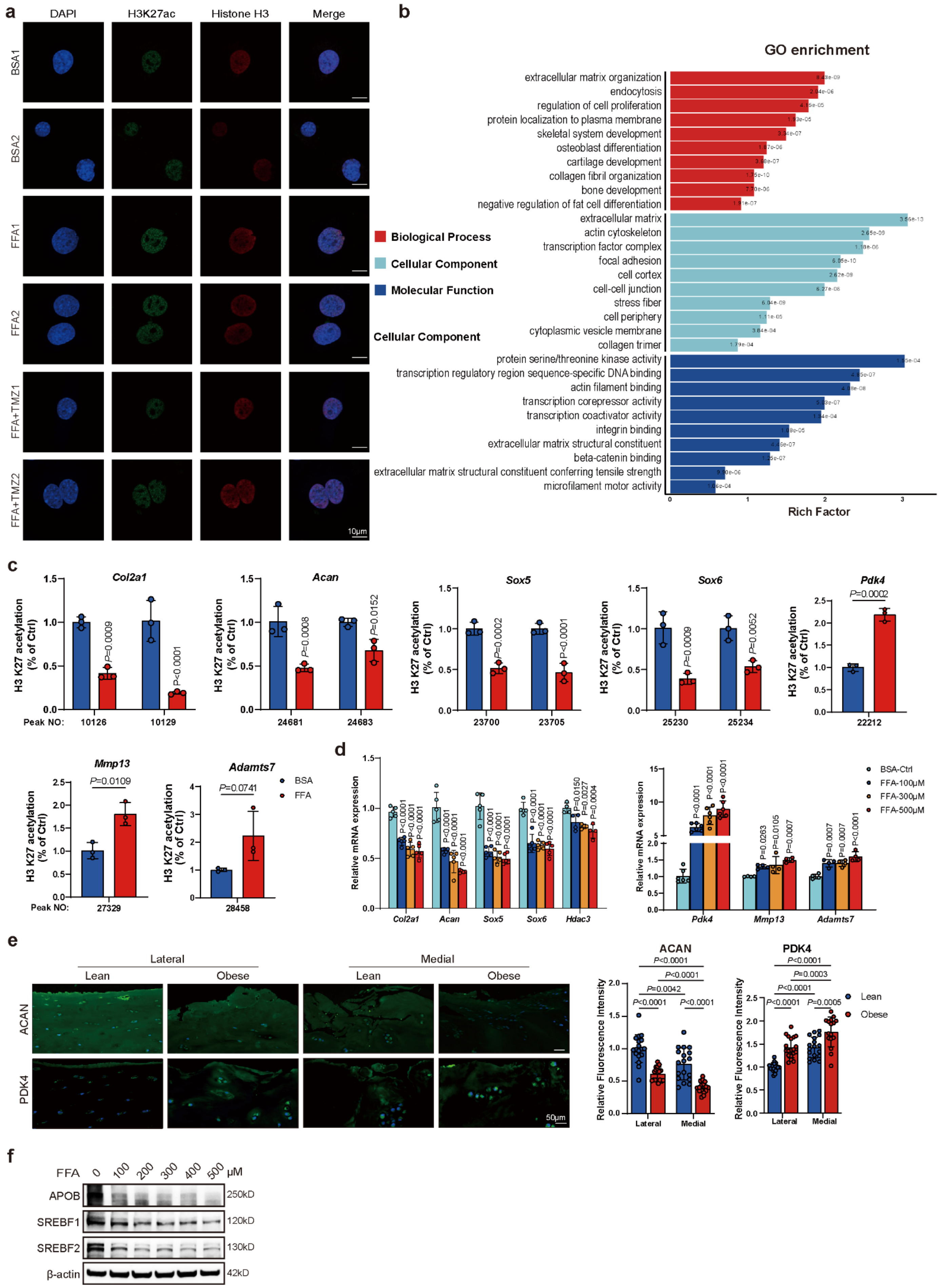

Figure S9. FFA epigenetically regulates cartilage ECM metabolism. (a) Representative confocal images of IF staining for H3K27ac and histone H3 in primary mouse chondrocytes treated with BSA, FFA, or FFA + TMZ. (b)

GO enrichment analysis of genes around significantly altered H3K27ac peaks (log_2 FC > 1, P < 0.01), as determined by a two-tailed Fisher's exact test followed by Benjamini–Hochberg (BH) correction. (c) Quantification of H3K27 acetylation at the gene locus, including *Col2a1*, *Acan*, *Sox5*, *Sox6*, *Pdk4*, *Mmp13*, and *Adamts7*, in BSA- and FFA-treated chondrocytes, assessed via CUT&Tag-qPCR (n = 3). Data are mean ± SD, based on unpaired two-tailed *t*-test. (d) mRNA expression levels of *Col2a1* (n = 5), *Acan* (n = 5), *Sox5* (n = 5), *Sox6* (n = 5), *Hdac3* (n = 4), *Pdk4* (n = 6), *Mmp13* (n = 4), and *Adamts7* (n = 4) in mouse chondrocytes treated with the indicated concentrations of FFA, as assessed via RT-qPCR. Data are mean ±SD, based on one-way ANOVA followed by Dunnett's multiple-comparisons test. (e) IF staining for ACAN and PDK4 in human knee cartilage indicated in Figure 1k (n = 18). Data are mean ±SD, based on two-tailed *t*-test or nonparametric two-tailed Mann–Whitney test. (f) Immunoblot detection of APOB, SREBF1 and SREBF2 in primary mouse chondrocytes treated with FFA. The western blot data are representative of three independent experiments. Source data are provided as a Source Data file.

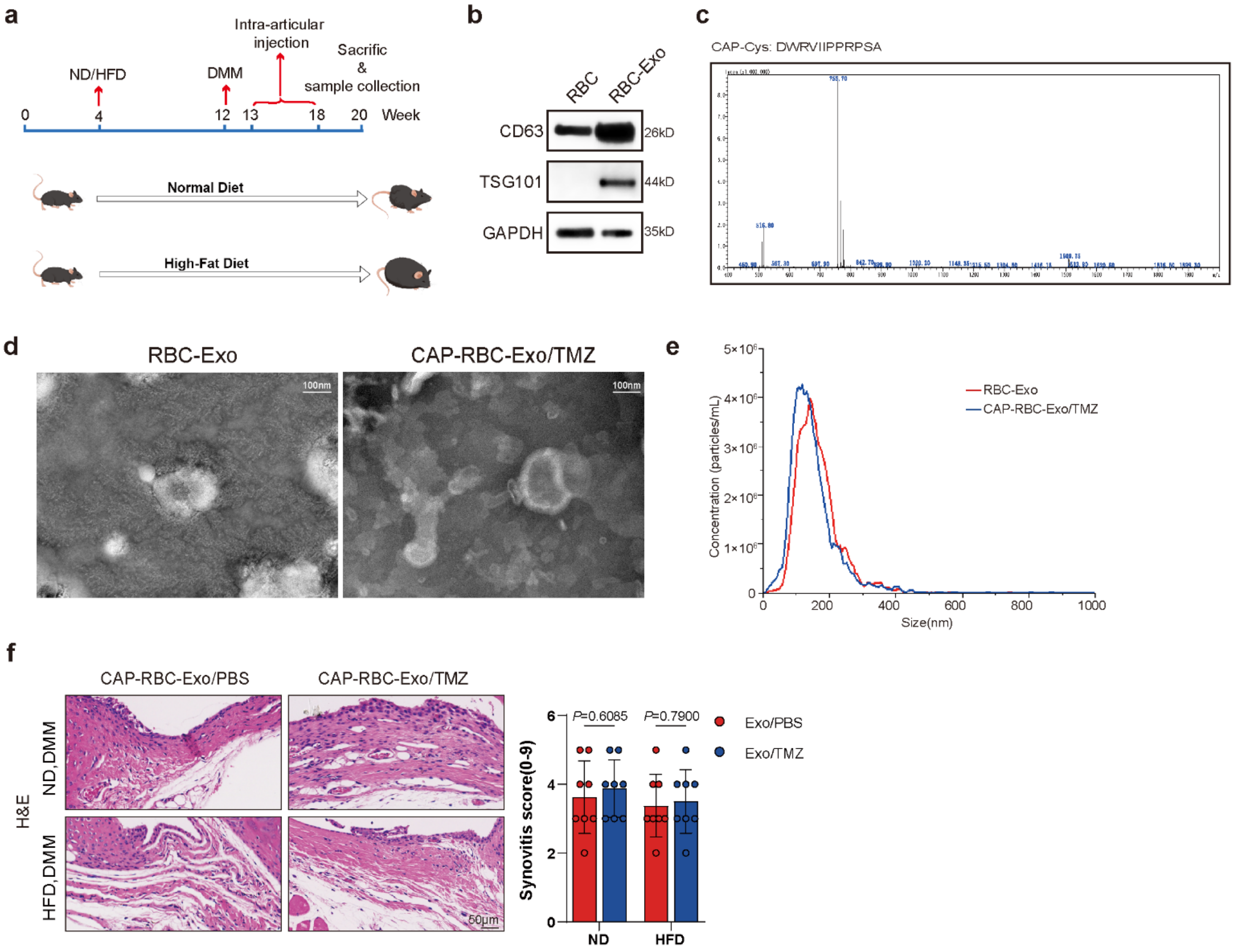

Figure S10. Isolation of RBC-exosomes (RBC-Exo) and production of CAP-RBC-Exo/TMZ. (a) Illustration of diet change and DMM surgery and arthrocentesis in male C57BL/6J mice, by figdraw.com. (b) Expression of marker proteins in whole cell lysates and lysates of purified exosomes examined by western blotting. (c) UPLC-MS/MS for identifying the chondrocyte-affinity peptide (CAP). (d) Representative transmission electron microscopy images of RBC-Exo and CAP-RBC-Exo/TMZ. (e) Size distribution of RBC-Exo and CAP-RBC-Exo/TMZ, determined by nanoparticle tracking analysis. (f) Hematoxylin and eosin staining (H&E) of synovial tissues in knee joints form mice indicated in Figure 6b. Synovitis scores was used to assess histological changes in the synovial tissues (n = 8). The data represent the mean ± SD, as determined by two-tailed *t*-test or nonparametric two-tailed Mann–Whitney test. Source data are provided as a Source Data file.

Supplementary Table 1. The medical records of patients underwent knee replacement surgery.

No.	Gender	Age(years)	Height(cm)	Body Weight(kg)	Body Mass Index	Kellgren &Lawrence Grade
1	Female	76	158	52	20.82	Ⅳ
2	Female	74	160	60	23.44	Ⅳ
3	Female	75	160	60	23.44	Ⅳ
4	Male	66	175	73	23.84	Ⅲ
5	Female	54	150	52	23.11	Ⅳ
6	Male	68	155	58	23.73	Ⅳ
7	Female	60	163	60	22.58	Ⅲ
8	Female	71	160	61	23.83	Ⅳ
9	Female	63	155	57	23.73	Ⅳ
10	Female	70	160	60	23.44	Ⅳ
11	Female	67	165	62	22.78	Ⅱ
12	Female	59	165	55	20.20	Ⅲ
13	Female	76	155	57	23.73	Ⅲ
14	Female	71	158	59	23.63	Ⅳ
15	Female	67	153	56	23.92	Ⅳ
16	Female	76	155	48	19.98	Ⅳ
17	Female	54	160	60	23.44	Ⅳ
18	Female	74	160	61	23.83	Ⅳ
19	Female	73	165	87	31.96	Ⅲ
20	Female	63	162	63	28.32	Ⅲ
21	Female	63	160	89	34.77	Left:IV Right:IV
22	Male	75	173	87	29.07	Ⅲ

23	Male	76	160	80	31.25	IV
24	Female	64	160	76	29.69	IV
25	Female	73	146	62	29.09	IV
26	Female	62	160	85	33.20	IV
27	Female	80	155	72	29.97	IV
28	Female	77	150	65	28.89	IV
29	Male	64	170	82	28.37	IV
30	Female	60	156	69	28.35	Left:IV Right:IV
31	Female	82	160	72	28.13	IV
32	Female	64	160	72	28.13	IV
33	Female	73	153	70	29.90	Left:Ⅲ Right:IV
34	Female	64	171	82	28.04	IV
35	Female	58	165	85	31.22	Left:IV Right:Ⅲ
36	Female	59	162	85	32.39	IV

Supplementary Table 2. The medical records and laboratory findings from patients underwent synovial fluid collection.

NO.	Age (years)	Gender	Height (cm)	Body weight (kg)	BMI	K & L Grade	Synovial fluid FFA (μM)	Synovial fluid TG (mM)	Synovial fluid FC (μM)	Synovial fluid TC (μM)
1	62	Female	152	45	19.48	0	575.58	0.27	721.68	2291.26
2	31	Male	175	65	21.22	0	721.90	0.32	724.82	2256.71
3	50	Female	158	53	21.23	0	597.87	0.29	642.53	2046.56
4	52	Female	164	58	21.56	0	589.15	0.23	619.77	2254.16
5	64	Male	166	60	21.77	0	440.89	0.18	693.48	2467.98
6	27	Male	177	69	22.02	0	331.40	0.27	672.21	2214.63
7	52	Female	160	57	22.27	0	370.16	0.32	636.26	2187.60
8	65	Female	165	65	23.88	0	597.87	0.31	649.62	2198.93
9	45	Female	157	68	27.59	0	395.35	0.34	568.98	2065.82
10	53	Female	153	68	29.05	0	739.34	0.25	689.53	2274.83
11	57	Female	153	70	29.90	0	608.53	0.24	680.13	2072.05
12	43	Female	150	80	35.56	0	548.45	0.21	645.00	2078.28
13	76	Female	150	36	16.00	1	518.41	0.30	635.44	2155.88
14	58	Female	163	45	16.94	1	472.87	0.22	563.38	2486.11
15	68	Male	160	49.5	19.34	1	844.96	0.18	679.47	2356.40
16	64	Female	168	56	19.84	1	588.18	0.25	632.64	2176.84
17	57	Male	168	57	20.20	1	435.08	0.25	575.74	2129.26
18	76	Male	175	62	20.24	1	422.48	0.31	573.11	2074.88
19	72	Female	150	46	20.44	1	427.33	0.33	562.22	2195.53
20	74	Male	170	60	20.76	1	672.48	0.19	652.01	2388.12
21	77	Female	148	48	21.91	1	482.56	0.23	679.63	2239.15
22	57	Male	158	55	22.03	1	582.36	0.33	629.50	2317.31
23	49	Female	160	57	22.27	1	636.63	0.32	704.53	2134.92
24	69	Female	155	54	22.48	1	689.92	0.24	675.51	2177.97
25	53	Female	162	60	22.86	1	393.41	0.26	635.27	2604.49
26	55	Female	168	65	23.03	1	416.67	0.25	704.20	2405.68
27	66	Female	167	70	25.10	1	395.35	0.29	610.87	2569.38
28	58	Female	172	75	25.35	1	594.96	0.34	722.67	2181.94
29	71	Female	160	65	25.39	1	434.11	0.28	562.88	2139.45
30	70	Female	150	60.5	26.89	1	728.68	0.20	575.41	2529.73
31	64	Female	158	69	27.64	1	511.63	0.27	722.18	2325.24
32	64	Male	170	80	27.68	1	730.62	0.31	724.98	2078.28
33	42	Female	169	80	28.01	1	672.48	0.32	584.98	2235.75
34	67	Female	158	70	28.04	1	477.71	0.26	603.94	2360.36
35	62	Female	163	75	28.23	1	570.74	0.17	573.76	2132.09
36	52	Male	178	97	30.61	1	738.37	0.32	722.18	2365.46
37	54	Female	155	75	31.22	1	760.66	0.26	588.77	2074.88
38	52	Female	165	85	31.22	1	720.72	0.27	713.44	2193.83
39	63	Female	158	85	34.05	1	900.90	0.31	571.29	2435.13
40	53	Female	156	85	34.93	1	559.11	0.22	602.38	2163.81
41	59	Female	164	57	21.19	2	409.88	0.26	609.30	2189.30

42	60	Female	160	55	21.48	2	641.64	0.24	605.26	2601.66
43	65	Male	173	65	21.72	2	697.18	0.23	686.56	2278.80
44	66	Female	155	54	22.48	2	736.43	0.24	634.61	2403.98
45	66	Female	155	54	22.48	2	583.33	0.27	579.21	2096.41
46	75	Female	156	55	22.60	2	555.23	0.24	610.04	2156.45
47	60	Female	155	55	22.89	2	409.88	0.34	585.80	2588.63
48	50	Male	180	75	23.15	2	814.92	0.30	712.78	2273.70
49	75	Female	160	60	23.44	2	467.05	0.34	688.37	2030.13
50	70	Female	160	60	23.44	2	402.13	0.33	616.14	2190.43
51	57	Female	160	60	23.44	2	506.51	0.28	614.33	2349.03
52	70	Female	153	55	23.50	2	592.05	0.25	635.27	2456.09
53	54	Male	165	65	23.88	2	859.50	0.27	669.24	2078.85
54	47	Female	155	57.5	23.93	2	656.01	0.19	675.18	2236.88
55	64	Female	157	60	24.34	2	553.29	0.19	585.31	2244.24
56	72	Female	162	65	24.77	2	422.48	0.32	616.97	2275.40
57	75	Female	155	60	24.97	2	618.22	0.24	681.86	2238.30
58	69	Female	163	67	25.22	2	692.83	0.19	711.29	2607.33
59	69	Female	167	72	25.82	2	468.99	0.22	704.04	2085.08
60	68	Male	176	80	25.83	2	660.85	0.19	681.78	2076.58
61	70	Female	153	63	26.90	2	622.09	0.23	701.07	2621.49
62	63	Female	162	70.6	26.90	2	623.55	0.28	630.00	2479.88
63	68	Female	150	62	27.56	2	638.57	0.28	585.97	2319.58
64	69	Female	157	68	27.59	2	387.60	0.32	583.00	2191.57
65	50	Female	162	75	28.58	2	947.95	0.32	670.56	2290.12
66	73	Female	146	62	29.09	2	347.87	0.22	659.68	2385.85
67	56	Female	160	75	29.30	2	739.74	0.20	698.60	2531.42
68	64	Female	160	76	29.69	2	424.42	0.35	562.06	2275.40
69	58	Female	160	76.5	29.88	2	847.85	0.30	557.11	2282.19
70	71	Female	158	75	30.04	2	970.97	0.26	675.10	2110.28
71	68	Male	178	97	30.60	2	420.54	0.18	668.75	2442.49
72	61	Female	155	75	31.22	2	516.47	0.24	633.46	2452.57
73	62	Male	165	85	31.22	2	344.96	0.25	585.80	2274.26
74	74	Male	160	80	31.25	2	758.72	0.18	566.01	2317.31
75	57	Female	163	85	31.99	2	639.53	0.21	584.98	2286.60
76	51	Male	175	100	32.65	2	877.91	0.33	731.25	2171.20
77	72	Female	155	42	17.48	3	505.81	0.18	672.21	2164.38
78	58	Male	175	58	18.94	3	973.97	0.26	574.75	2596.00
79	66	Male	170	55	19.03	3	683.90	0.24	650.36	2335.44
80	66	Female	150	45	20.00	3	531.98	0.34	603.94	2494.61
81	67	Male	161	56.5	21.80	3	830.79	0.29	581.35	2255.57
82	65	Female	155	52.5	21.85	3	425.39	0.30	652.42	2366.59
83	85	Female	156	55	22.60	3	584.30	0.34	623.65	2275.96
84	69	Female	157	57	23.12	3	822.67	0.22	659.84	2272.57
85	58	Female	178	74	23.40	3	529.07	0.23	645.99	2129.56
86	74	Malae	165	64	23.51	3	827.55	0.24	625.21	2435.32
87	74	Female	149	53	23.87	3	674.42	0.27	641.05	2487.67
88	73	Female	165	65	23.88	3	847.87	0.20	596.69	2303.26

89	73	Female	165	67.5	24.79	3	626.45	0.31	652.51	2248.24
90	74	Female	163	67	25.22	3	562.02	0.21	610.21	2185.48
91	72	Female	160	65	25.39	3	500.00	0.34	694.80	2122.42
92	66	Female	160	66.2	25.90	3	766.47	0.20	605.92	2488.86
93	61	Female	158	68	27.24	3	669.57	0.21	571.95	2487.07
94	78	Female	154	65	27.41	3	626.94	0.31	601.63	2487.07
95	74	Female	164	74	27.50	3	566.86	0.25	625.21	2121.83
96	75	Male	176	86	27.76	3	524.22	0.31	657.54	2124.21
97	58	Female	160	72	28.13	3	905.91	0.32	650.61	2124.21
98	64	Female	164	78	29.00	3	843.02	0.34	624.39	2168.23
99	58	Female	165	79	29.02	3	713.18	0.26	642.53	2171.20
100	75	Male	173	87	29.07	3	721.90	0.24	719.04	2196.19
101	76	Male	165	80	29.38	3	1046.05	0.23	614.33	2168.82
102	79	Male	182	100	30.19	3	584.58	0.31	720.86	2597.12
103	65	Male	170	87.5	30.28	3	796.80	0.32	562.88	2196.19
104	63	Female	156	77.5	31.80	3	554.26	0.30	695.63	2168.23
105	50	Female	153	75	32.04	3	1100.10	0.33	596.52	2663.15
106	62	Female	160	85	33.20	3	505.81	0.24	591.08	2475.18
107	55	Male	170	62	21.45	4	894.89	0.26	610.54	2347.28
108	54	Female	150	52	23.11	4	784.88	0.22	711.46	2149.79
109	69	Male	178	74	23.40	4	404.07	0.34	731.74	2347.28
110	83	Female	165	65	23.88	4	804.26	0.23	647.81	2470.42
111	65	Female	163	65	24.46	4	600.78	0.23	683.67	2410.04
112	75	Male	169	74	25.91	4	557.17	0.34	595.70	2169.42
113	77	Male	161	71	27.39	4	631.78	0.26	567.50	2458.52
114	65	Male	175	86	28.08	4	642.44	0.25	587.12	2390.71
115	70	Female	156	66	28.12	4	860.47	0.34	689.53	2274.11
116	70	Male	173	87	29.07	4	721.90	0.30	612.52	2149.19
117	71	Female	158	74	29.64	4	875.00	0.31	673.12	2332.71
118	76	Male	165	73	31.25	4	715.12	0.30	696.95	2205.70
119	52	Female	155	80	33.30	4	1137.14	0.32	579.21	2607.83
120	63	Female	160	89	34.77	4	675.39	0.22	624.72	2588.79

BMI= Body Mass Index; K & L Grade=Kellgren & Lawrence Grade; FFA=free fatty acids; TG=triglyceride; FC=free cholesterol; TC= total cholesterol

Supplementary Table 3. Oligonucleotide and primer list.

REAGENT or RESOURCE	SOURCE	IDENTIFIER
**si/shRNA for gene knockdown**		
Hadha si/shRNA #1: CCAATACAGAATAGCAACATT	This paper	N/A
Hadha si/shRNA #2: GGGATTCCATCTTCAGCAATT	This paper	N/A
Hadhb si/shRNA #1: GTACAGTTATTCAGGAAGTTT	This paper	N/A
Hadhb si/shRNA #2: CTGTCACCATGGCTTGTATTT	This paper	N/A
TRIM9 si/shRNA #1: GACAAGATGAGCCTATACATT	This paper	N/A
TRIM9 si/shRNA #2: GACCTGTAGTAGCTATGATTT	This paper	N/A
**gRNA for gene knockout**		
Hadha sgRNA: AAGTCCACAAAGCCTATTG	This paper	N/A
Hadhb sgRNA: TACCTGGGGCAGCTCGTAGC	This paper	N/A
**Primers for RT-qPCR**		
β-actin-F: GGCTGTATTCCCCTCCATCG	This paper	N/A
β-actin-R: CCAGTTGGTAACAATGCCATGT	This paper	N/A
Col2a1-F: TGGCTTAGGGCAGAGAGAGA	This paper	N/A
Col2a1-R: CGTCGTGCTGTCTCAAGGT	This paper	N/A
Acan-F: CCTGCTACTTCATCGACCCC	This paper	N/A
Acan-R: AGATGCTGTTGACTCGAACCT	This paper	N/A
Sox9-F: TAATTCCCCAGGCTCTTGGAT	This paper	N/A
Sox9-R: GCAGCCGGGATTTAAGGCTC	This paper	N/A
Sox5-F: CCCGTGATCCAGAGCACTTAC	This paper	N/A
Sox5-R: CCGCAATGTGGTTTTCGCT	This paper	N/A
Sox6-F: GGTCATGTTTCCCACCCACAA	This paper	N/A
Sox6-R: TTCAGAGGGGTCCAAATTCCT	This paper	N/A
Hdac3-F: GCCAAGACCGTGGCGTATT	This paper	N/A
Hdac3-R: GTCCAGCTCCATAGTGGAAGT	This paper	N/A
Pdk4-F: AGGGAGGTCGAGCTGTTCTC	This paper	N/A
Pdk4-R: GGAGTGTTCACTAAGCGGTCA	This paper	N/A
Mmp13-F: CAAGCAGTTCCAAAGGCTACA	This paper	N/A
Mmp13-R: TAGGGCTGGGTCACACTTCT	This paper	N/A
Adamts7-F: AGTGTCCCAACCTGCCATTTG	This paper	N/A
Adamts7-R: CCTAGAGCCTTGGTGCTTGTA	This paper	N/A
Lipe-F: CCAGCCTGAGGGCTTACTG	This paper	N/A
Lipe-R: CTCCATTGACTGTGACATCTCG	This paper	N/A
Cd36-F: GGAGCCATCTTTGAGCCTTCA	This paper	N/A
Cd36-R: GAACCAAACTGAGGAATGGATCT	This paper	N/A
Fatp5-F: GTTCTCCCGTCCAAGACCATT	This paper	N/A
Fatp5-R: GCTCCGTACAGAGTGTAGCAAG	This paper	N/A
Fabp3-F: ACCTGGAAGCTAGTGGACAG	This paper	N/A
Fabp3-R: TGATGGTAGTAGGCTTGGTCAT	This paper	N/A
Apob-F: GAGACAGGAGCAGTAATACAGCC	This paper	N/A
Apob-R: GGCCACCCAGTCTATCTCTTTG	This paper	N/A
Srebf1-F: GATGTGCGAACTGGACACAG	This paper	N/A
Srebf1-R: CATAGGGGGCGTCAAACAG	This paper	N/A
Srebf2-F: GCAGCAACGGGACCATTCT	This paper	N/A
Srebf2-R: CCCCATGACTAAGTCCTTCAACT	This paper	N/A

Esr1-F: CCTCCCGCCTTCTACAGGT	This paper	N/A
Esr1-R: CACACGGCACAGTAGCGAG	This paper	N/A
**Primers for CUT&Tag-qPCR**		
DNA Spike-in-F: GCCTTCTTCCCATTTCTGATCC	This paper	N/A
DNA Spike-in-R: CACGAATCAGCGGTAAAGGT	This paper	N/A
Col2a1-peak10126-F: ACTGAGCTCGAAACGTCCTG	This paper	N/A
Col2a1-peak10126-R: GACCTCTGTCGCAGCATCTT	This paper	N/A
Col2a1-peak10129-F: TCAGATAGAGCACCTGGCCT	This paper	N/A
Col2a1-peak10129-R: AAAGGCCTCTGATGCTTCCC	This paper	N/A
Acan-peak24681-F: CAGTGGCTCTCCAGCTTCTC	This paper	N/A
Acan-peak24681-R: CTCCTTCTCCTCTCTGCCCT	This paper	N/A
Acan peak24683-F: ATTCCCAGAGTAGGGGGTCG	This paper	N/A
Acan-peak24683-R: GGGTGTTTCCCTGGTATCGG	This paper	N/A
Sox5-peak23700-F: CCAGGTGCGGTGTCATATCT	This paper	N/A
Sox5-peak23700-R: GAGGTGGTTCAAACAGCCGA	This paper	N/A
Sox5-peak23705-F: TGTGACAGTCAGCTCGTCTC	This paper	N/A
Sox5-peak23705-R: GGATCGGTTCCAAGCCCAA	This paper	N/A
Sox6-peak25230-F: CAGAGGAGGCCAGCATTTGA	This paper	N/A
Sox6-peak25230-R: TTCCGTGGACATGAGAAGCC	This paper	N/A
Sox6-peak25234-F: AACCCCCTGTCTATCGCTGT	This paper	N/A
Sox6-peak25234-R: AGTCTGCTGGCTGTGAAGAT	This paper	N/A
Pdk4-peak22212-F: GCAAGGACCACCCTACAGTT	This paper	N/A
Pdk4-peak22212-R: ACCCTCAGCAAATCGCTCTT	This paper	N/A
Mmp13-peak27329-F: GAAGCTCAAGGATGCGCCTA	This paper	N/A
Mmp13-peak27329-R: AACCAGTTGGCCTCAGTGTT	This paper	N/A
Adamts7-peak28458-F: AGCAACTTCTGCTCTGCCAT	This paper	N/A
Adamts7-peak28458-R: GTGCTGAGTGTCTGTGGGAA	This paper	N/A
**Primers for mice identification**		
Acan-CreER^{T2}-F WT: GTTATATTCCGGAGCCCACA	This paper	N/A
Acan-CreER^{T2}-F Mut: CTCCAGACTGCCCTTGGGAAAA		
Acan-CreER^{T2}-R Common: AAAAGCGACAAGAAGACACCA	This paper	N/A
Col2-CreER^{T2}-F: GAACGCACTGATTTCGACCA	This paper	N/A
Col2-CreER^{T2}-R: GCTAACCAGCGTTTTCGTTC	This paper	N/A
Hadha$^{fl/fl}$-F: GTGAAGAAACCACCTGACATTGG	This paper	N/A
Hadha$^{fl/fl}$-R: ACTGGTCCTTATGAATGGAGTAGG	This paper	N/A
Hadhb$^{fl/fl}$-F: CTAAATTATCCCACCACGACCC	This paper	N/A
Hadhb$^{fl/fl}$-R: CCAGAGGACAGTGCTCATTTAGTA	This paper	N/A
Hadha$^{CKI/CKI}$ -Mut-F: TCAGACTGGTCCGAATCCAC	This paper	N/A
Hadha$^{CKI/CKI}$-Mut-R: TGCACAAGGATAAAGGAAGGCAAC	This paper	N/A
Hadha$^{CKI/CKI}$ -WT-F: CGAGGTGTAAGTTGATTTGAGTGA	This paper	N/A
Hadha$^{CKI/CKI}$-WT-R: TGCACAAGGATAAAGGAAGGCAAC	This paper	N/A

Supplementary Table 4. The OA cartilage histopathology grade.

**Grade (key feature)**	**Associated criteria (tissue reaction)**
Grade 0: surface intact, cartilage morphology intact	Matrix: normal architecture
	Cells: intact, appropriate orientation
Grade 1: surface intact	Matrix: superficial zone intact, oedema and/or superficial fibrillation (abrasion), focal superficial matrix condensation
	Cells: death, proliferation (clusters), hypertrophy, superficial zone
	Reaction must be more than superficial fibrillation only
Grade 2: surface discontinuity	As above
	+Matrix discontinuity at superficial zone (deep fibrillation)
	±Cationic stain matrix depletion (Safranin O or Toluidine Blue) upper 1/3 of cartilage
	±Focal perichondronal increased stain (mid zone)
	±Disorientation of chondron columns
	Cells: death, proliferation (clusters), hypertrophy
Grade 3: vertical fissures (clefts)	As above
	Matrix vertical fissures into mid zone, branched fissures
	±Cationic stain depletion (Safranin O or Toluidine Blue) into lower 2/3 of cartilage (deep zone)
	± New collagen formation (polarized light microscopy, Picro Sirius Red stain)
	Cells: death, regeneration (clusters), hypertrophy, cartilage domains adjacent to fissures
Grade 4: erosion	Cartilage matrix loss: delamination of superficial layer, mid layer cyst formation
	Excavation: matrix loss superficial layer and mid zone
Grade 5: denudation	Surface: sclerotic bone or reparative tissue including fibrocartilage within denuded surface. Microfracture with repair limited to bone surface
Grade 6: deformation	Bone remodelling (more than osteophyte formation only). Includes: microfracture with fibrocartilaginous and osseous repair extending above the previous surface

Supplementary Table 5. The recommended semi-quantitative scoring system (OARSI score).

Grade	Osteoarthritic damage
0	Normal
0.5	Loss of Safranin-O without structural changes
1	Small fibrillations without loss of cartilage
2	Vertical clefts down to the layer immediately below the superficial layer and some loss of surface lamina
3	Verical clefts/erosion to the calcified cartilage extending to <25% of the articular surface
4	Vertical clefts/erosion to the calcified cartilage extending to 25–50% of the articular surface
5	Vertical clefts/erosion to the calcified cartilage extending to 50–75% of the articular surface
6	Vertical clefts/erosion to the calcified cartilage extending >75% of the articular surface

Supplementary Table 6. The Mankin Histology histopathology grading system.

	Grade
**I. Structure**	
a. Normal	0
b. Surface irregularities	1
c. Pannus and surface irregularities	2
d. Clefts to transitional zone	3
e. Clefts to radial zone	4
f. Clefts to calcified zone	5
g. Complete disorganisation	6
**II. Cells**	
a. Normal	0
b. Diffuse hypercellularity	1
c. Cloning	2
d. Hypocellularity	3
**III. Safranin-O staining**	
a. Normal	1
b. Slight reduction	2
c. Moderate reduction	3
d. Severe reduction	4
e. No dye noted	5
**IV. Tidemark integrity**	
a. Intact	0
b. Crossed by blood vessels	1

Mankin HHGS score is the sum of structure, cells, Safranin-O staining and tidemark integrity.

Supplementary Table 7. Multiple reaction monitoring parameters

Compound	Precursor (m/z)	Product(m/z)	CE(V)	RF Lens (V)	Polarity	IS
Acetyl-CoA	810	303.05	31.14	102	+	4-CPA
$^{13}C_2$-Acetyl-CoA	812	305.05	30.49	143	+	4-CPA
4-CPA	200	154	13	59	+	

Supplementary Table 8. Key resources table.

REAGENT or RESOURCE	SOURCE	IDENTIFIER
Antibodies		
Rabbit monoclonal Anti- HADHA	Abcam	Cat#ab203114
Rabbit polyclonal Anti-HADHB	Abcam	Cat#ab230667
Rabbit monoclonal Anti-SOX9	Abcam	Cat#ab185230
Rabbit monoclonal Anti-MMP3	Abcam	Cat#ab52915
Rabbit polyclonal Anti-MMP13	Abcam	Cat#ab39012
Rabbit polyclonal Anti- Acetyl Lysine	Abcam	Cat#ab21623
Rabbit monoclonal Anti-Ubiquitin	Abcam	Cat#ab134953
Rabbit monoclonal Anti-TSG101	Abcam	Cat#ab125011
Rabbit monoclonal Anti-Phospho-ACC (Ser79)	Cell Signaling Technology	Cat# 11818
Rabbit monoclonal Anti-ACC	Cell Signaling Technology	Cat #3676
Rabbit monoclonal Anti-Phospho-AMPKα (Thr172)	Cell Signaling Technology	Cat# 2531
Rabbit monoclonal Anti-AMPKα	Cell Signaling Technology	Cat#5831
Rabbit monoclonal Anti- Phospho-AMPK Substrate Motif	Cell Signaling Technology	Cat# 5759
Mouse monoclonal Anti-Beta Actin	Proteintech	Cat#66009-1-Ig
Mouse monoclonal Anti-GAPDH	Proteintech	Cat#60004-1-Ig
Mouse monoclonal Anti-CD63	Proteintech	Cat#67605-1-Ig
Rabbit polyclonal Anti-TRIM9	Proteintech	Cat#10786-1-AP
Rabbit polyclonal anti-PDK4	Proteintech	Cat#12949-1-AP
Mouse monoclonal anti-ACAN	Millipore	Cat#C8035
Rabbit polyclonal Anti-Acetyl-HADHA at Lysine 728	this paper	N/A
Mouse Monoclonal Anti-Histone H3	Beyotime Biotechnology	Cat#Cat: AF0009
Rabbit polyclonal Anti-Histone H3K27ac	Active Motif	Cat#39133
Rabbit polyclonal Anti-COL2A1	Bioss	Cat#bs-10589R
Mouse monoclonal Anti-FLAG HRP	Sigma-Aldrich	Cat#A8592
Mouse monoclonal Anti-HA HRP	GenScript	Cat#A01296
Anti-DYKDDDDK IP Resin	GenScript	Cat#L00425
Anti-HA magnetic beads	Selleck chemicals	Cat#B26202
Goat Anti-Mouse HRP	FUDE Biotech	Cat#FDM007
Goat Anti-Rabbit HRP	FUDE Biotech	Cat#FDR007
Alexa Fluor 594 goat anti-mouse IgG	FUDE Biotech	Cat#FD0143
Alexa Fluor 488 goat anti-rabbit IgG	FUDE Biotech	Cat#FD0136
Biological Samples		
Human cartilage samples, see Table S1	Sir Run Run Shaw Hospital,<br>Zhejiang University School of Medicine	N/A
Human synovial fluid, see Table S2	Sir Run Run Shaw Hospital,<br>Zhejiang University School of Medicine	N/A

Healthy adult peripheral blood	Volunteers from Sir Run Run Shaw Hospital, Zhejiang University School of Medicine	N/A
Chemicals, Peptides, and Recombinant Proteins		
IBMX	Sigma-Aldrich	Cat#I7018
Palmitic acid	Sigma-Aldrich	Cat#P0500
Oleic acid	Sigma-Aldrich	Cat#O1008
Acetyl-1,2-$^{13}C_2$ coenzyme A lithium salt	Sigma-Aldrich	Cat#658650
DL-β-Hydroxybutyryl coenzyme A lithium salt	Sigma-Aldrich	Cat#H0261
NAD	Sigma-Aldrich	Cat#NAD98-RO
1-MPMS	Sigma-Aldrich	Cat#M8640
FCCP	TargetMOI	Cat#T6834
Oligomycin A	TargetMOI	Cat#T6323
Rotenone	TargetMOI	Cat#T2970
Trichostatin A	TargetMOI	Cat#T6270
A-769662	TargetMOI	Cat#T2468
Dorsomorphin dihydrochloride	TargetMOI	Cat#T6146
Cycloheximide (CHX)	Selleck chemicals	Cat#S7418
Etomoxir sodium salt	Selleck chemicals	Cat#S8244
Trimetazidine dihydrochloride	Selleck chemicals	Cat#S4543
MG132	Selleck chemicals	Cat#S2619
Chloroquine	Selleck chemicals	Cat#S6999
Recombinant Human Insulin	Solarbio	Cat#I8830
Dexamethasone	Solarbio	Cat#D8040
Nile red	MCE	Cat#HY-D0718
BODIPY 493/503	MCE	Cat#HY-D1614
WST-8	MCE	Cat#HY-D0831
UK5099	MCE	Cat#HY-15475
BPTES	MCE	Cat#HY-12683
Ca2+ ionophore	Abcam	Cat#ab120370
Antimycin A	Abcam	Cat#ab141904
Nicotinamide	Sangon Biotech	Cat#A510659
Recombinant Human TNF-alpha	R&D systems	Cat#10291-TA
Recombinant Human IL-1 beta	R&D systems	Cat#201-LB
Fatty Acid Mix (U-13C, 98%)	Cambridge Isotope Laboratories	Cat#CLM-8455-PK
3×FALG peptide	YEASEN	Cat#20571ES
SYBR Green Master Mix	Vazyme Biotech	Cat#Q711-02
TRIzol	Takara	Cat#9109
Collagenase II	Sigma-Aldrich	Cat#C2-BIOC
Antifade mounting medium with DAPI	Meilunbio	Cat#MA0222
FBS	Cellmax	Cat#SA101.02
NBCS	Gibco	Cat#16010159

Critical Commercial Assays		
Free Fatty Acid Uptake Assay	Abcam	Cat#ab176768
Acetyl-CoA ELISA Kit	Elabscience	Cat#E-EL-0125c
ATP Chemiluminescence Assay Kit	Elabscience	Cat#E-BC-F002
Seahorse XFe96 FluxPaks	Agilent	Cat#103793-100
Seahorse XF Palmitate-BSA FAO Substrate	Agilent	Cat#102720-100
Nonesterified Free fatty acids (NEFA) assay kit	Aidisheng Biotech	Cat#DS-W-ZF001-96
Triglyceride (TG) Assay Kit	Aidisheng Biotech	Cat#ADS-W-ZF013
Total Cholesterol Assay Kit	Meimian Biotech	Cat#MM-50977H1
Free Cholesterol Assay Kit	Meimian Biotech	Cat#MM-63462H1
Hyperactive Universal CUT&Tag Assay Kit for Illumina Pro	Vazyme Biotech	Cat#TD904-02
Mouse Direct PCR kit	Bimake	Cat# B40015
Super Vision IHC kit	BOSTER	Cat#SV0004
BCA protein assay kit	Meilunbio	Cat#MA0082
Special deparaffinized Reagent Kit for Lipid Staining	HePeng Biotech	Cat#HPBIO-JM4801
SteadyPure RNA extraction kit	Accurate Biotechnology	Cat#AG21017
Experimental Models: Cell Lines		
C28/I2	Sigma-Aldrich	Cat#SCC043
3T3-L1	ATCC	Cat#CL-173
HEK293T	ATCC	Cat#CRL-11268
HADHA-KO C28/I2	this paper	N/A
HADHB-KO C28/I2	this paper	N/A
Primary mouse chondrocytes	this paper	N/A
Experimental Models: Organisms/Strains		
Mouse: C57BL/6	Hangzhou QiZhen Laboratory Animal Technology Center	N/A
Mouse: Acan-CreER^{T2}	Prof. Ximei Wu from Zhejiang University School of Medicine	N/A
Mouse: Col2-CreER^{T2}	Jackson Laboratory	N/A
Hadha$^{fl/fl}$	Cyagen Biology Technology	N/A
Hadhb$^{fl/fl}$	Cyagen Biology Technology	N/A
Hadha$^{CKI/CKI}$ (p. K728R Conditional Knock in)	Cyagen Biology Technology	N/A
Oligonucleotides		
si/shRNA for gene knockdown, see Table S3	this paper	N/A
Primers for RT-qPCR, see Table S3	this paper	N/A
Primers for CUT&Tag-qPCR, see Table S3	this paper	N/A
gRNA for gene knockout, see Table S3	this paper	N/A
Primers for mice identification, see Table S3	this paper	N/A
Recombinant DNA		
PLKO.1 puro	Addgene	Cat#8453

PEP-KO	this paper	N/A
pLVX-HADHA-Flag	this paper	N/A
pLVX-HADHA K728R-Flag	this paper	N/A
pLVX-HADHB-Flag	this paper	N/A
pLVX -HADHA-HA	this paper	N/A
pcDNA3.1-SOX9-Flag	this paper	N/A
pcDNA3.1-SOX9 T25A-Flag	this paper	N/A
pcDNA3.1-SOX9 T87A-Flag	this paper	N/A
pcDNA3.1-SOX9 S98A-Flag	this paper	N/A
pcDNA3.1-SOX9 S136A-Flag	this paper	N/A
pcDNA3.1-SOX9 S138A-Flag	this paper	N/A
pcDNA3.1-SOX9 T193A-Flag	this paper	N/A
pcDNA3.1-SOX9 ST-A-Flag (T25A, T87A, S98A, S136A, S138A, and T193A)	this paper	N/A
pcDNA3.1-SOX9 ST-D-Flag (T25D, T87D, S98D, S136D, S138D, and T193D)	this paper	N/A
pLVX-HA-ZNF598	this paper	N/A
pLVX-HA-TRIM9	this paper	N/A
pLVX-HA-RLIM	this paper	N/A
Software and Algorithms		
GraphPad Prism 9.2.1	https://www.graphpad.com/	N/A
R 4.1.2	https://www.r-project.org/	N/A
Image J v1.50	https://imagej.net/	N/A
Wave 2.4.0	https://www.agilent.com/	N/A
IGV_2.16.2	https://igv.org/	N/A
NIS-Elements Viewer 4.20	https://www.microscope.healthcare.nikon.com/	N/A
Other		
Safranin O	Sigma-Aldrich	Cat#S8884
Fast Green	Sigma-Aldrich	Cat#F7252
Tamoxifen	Sigma-Aldrich	Cat#T2859
Alcian Blue 8 GS	Solarbio	Cat#IA2460
Nuclear fast red	Solarbio	Cat# G1321
Neutral balsam	Solarbio	Cat#G8590
Protease Inhibitor Cocktail	TargetMOI	Cat#C0001
PMSF	FUDE Biotech	Cat#FD0100
Bovine Serum Albumin (fatty acids free)	Meilunbio	Cat#MB0094-1
HFD with 60% of the calories from fat	Research Diets	Cat#D12492
Normal diet	XIETONG BIO-ENGINEERING CO., LTD.	Cat#XTCON50J
Polybrene	Tsingke	Cat#40804ES76
Protein A/G magnetic beads	Thermo Fisher Scientific	Cat#88802
Lipofectamine™ RNAiMAX	Thermo Fisher Scientific	Cat#13778030

Hieff Trans® Liposomal Transfection Reagent	YEASEN	Cat#40802ES03
Hieff qPCR SYBR Green Master Mix	YEASEN	Cat#11201
Evo MMLV RT Premix	Accurate Biotechnology	Cat#AG11706
Penicillin-Streptomycin	Biosharp	Cat#BL505A
Amersham Protran 0.45 NC membranes	Cytiva	Cat#10600002
5 × Loading buffer	FUDE Biotech	Cat#FD002
Dura ECL	FUDE Biotech	Cat#FD8020
3,3'-diaminobenzidine (DAB)	BOSTER	Cat#AR1027
Hematoxylin	Beyotime	Cat#C0107
Poly-D-lysine	Beyotime	Cat#ST508
