# Supplementary material for: Chondrocyte fatty acid oxidation drives osteoarthritis via SOX9 degradation and epigenetic regulation
Source: Nat Commun. 2025 May 27;16:4892. doi: 10.1038/s41467-025-60037-4 (PMC12117060; doi:10.1038/s41467-025-60037-4)
Supplement: Supplementary file 2 — Description of Additional Supplementary Files [file 41467_2025_60037_MOESM2_ESM.pdf]

Description of Additional Supplementary Files

Supplementary Data 1. Lipidomic data, related to Figures S1i and S1j.

Supplementary Data 2. Proteomics data, related to Figures S3g and S3h.

Supplementary Data 3. Acetylome data, related to Figures 2c,2d and S3j.

Supplementary Data 4. CUT&Tag-sequencing data, related to Figures 5c,5d,5e and S9b.
