# Supplementary material for: Chondrocyte fatty acid oxidation drives osteoarthritis via SOX9 degradation and epigenetic regulation
Source: Nat Commun. 2025 May 27;16:4892. doi: 10.1038/s41467-025-60037-4 (PMC12117060; doi:10.1038/s41467-025-60037-4)
Supplement: Supplementary file 7 — Reporting Summary [file 41467_2025_60037_MOESM7_ESM.pdf]

nature portfolio

Corresponding author(s): Shunwu Fan, Haitao Zhang, Shuying Shen

Last updated by author(s): Apr 10, 2025

Reporting Summary

Nature Portfolio wishes to improve the reproducibility of the work that we publish. This form provides structure for consistency and transparency in reporting. For further information on Nature Portfolio policies, see our Editorial Policies and the Editorial Policy Checklist.

Statistics

For all statistical analyses, confirm that the following items are present in the figure legend, table legend, main text, or Methods section.

n/a	Confirmed	
☐	☒	The exact sample size (*n*) for each experimental group/condition, given as a discrete number and unit of measurement
☐	☒	A statement on whether measurements were taken from distinct samples or whether the same sample was measured repeatedly
☐	☒	The statistical test(s) used AND whether they are one- or two-sided<br>*Only common tests should be described solely by name; describe more complex techniques in the Methods section.*
☒	☐	A description of all covariates tested
☐	☒	A description of any assumptions or corrections, such as tests of normality and adjustment for multiple comparisons
☐	☒	A full description of the statistical parameters including central tendency (e.g. means) or other basic estimates (e.g. regression coefficient) AND variation (e.g. standard deviation) or associated estimates of uncertainty (e.g. confidence intervals)
☐	☒	For null hypothesis testing, the test statistic (e.g. *F*, *t*, *r*) with confidence intervals, effect sizes, degrees of freedom and *P* value noted<br>*Give P values as exact values whenever suitable.*
☒	☐	For Bayesian analysis, information on the choice of priors and Markov chain Monte Carlo settings
☒	☐	For hierarchical and complex designs, identification of the appropriate level for tests and full reporting of outcomes
☒	☐	Estimates of effect sizes (e.g. Cohen's *d*, Pearson's *r*), indicating how they were calculated

Our web collection on statistics for biologists contains articles on many of the points above.

Software and code

Policy information about availability of computer code

Data collection	1. Image analysis:Image J v1.50.<br>2. qRT-PCR analysis: QuantStudio 5 Flex Real-Time PCR System (Thermo Fisher Scientific)<br>3. Western blot analysis: Amersham Imager (USA) and BIO-RAN ChemiDoc XRS+system v3.4.2<br>4. Immunoflourescence analysis: Fluorescence microscope (Olympus, Japan) and Nikon A1R confocal microscope (Japan)<br>5. Immunohistochemistry analysis: Microscope (Nikon, Japan)<br>6. Computational modeling and molecular docking: AutoDock Tools 1.5.6., HDCOK., MOPAC., and AutoDock 4.2.6..<br>7.Extracellular flux analysis: Seahorse XFe96 Extracellular Flux Analyzer (Agilent)<br>8.HADHA enzyme activity assay: Synergy H1 Multimode Reader (Agilent).<br>9. Statistical analysis: GraphPad Prism v9.2.1.
Data analysis	Data analysis was performed by using GraphPad Prism v9.2.1. Images were analyzed by Image J sftware (v1.50.)

For manuscripts utilizing custom algorithms or software that are central to the research but not yet described in published literature, software must be made available to editors and reviewers. We strongly encourage code deposition in a community repository (e.g. GitHub). See the Nature Portfolio guidelines for submitting code & software for further information.

Data

Policy information about availability of data

All manuscripts must include a data availability statement. This statement should provide the following information, where applicable:

- Accession codes, unique identifiers, or web links for publicly available datasets
- A description of any restrictions on data availability
- For clinical datasets or third party data, please ensure that the statement adheres to our policy

All data needed to evaluate the conclusions in the paper are present in the paper and/or the Supplementary Materials. The lipidomic data generated in this study have been deposited in the OMIX database under OMIX008076 [https://ngdc.cncb.ac.cn/omix/release/OMIX008076]. The proteomics and acetylome data generated in this study have been deposited in the iProX database under IPX0011615000 [https://www.iprox.cn/page/project.html?id=IPX0011615000]. The CUT&Tag-seq data generated in this study have been deposited in the GSA database under CRA020878 [https://ngdc.cncb.ac.cn/gsa/browse/CRA020878]. The electronic structure calculations data generated in this study have been deposited in the Figshare database under DOI [https://doi.org/10.6084/m9.figshare.28741640.v1]. Source data are provided with this paper.

Research involving human participants, their data, or biological material

Policy information about studies with human participants or human data. See also policy information about sex, gender (identity/presentation), and sexual orientation and race, ethnicity and racism.

Reporting on sex and gender	Human cartilage samples were collected from 36 patients (female 31 and male 5) with OA and received total knee joint replacement.<br>Human synovial fluid samples were collected from 120 patients(female 86 and male 34) diagnosed with OA and underwent arthrocentesis or arthroplasty.<br>Human red blood cells were isolated from peripheral blood obtained from two healthy male volunteers aged between 22 and 28 years.
Reporting on race, ethnicity, or other socially relevant groupings	*Please specify the socially constructed or socially relevant categorization variable(s) used in your manuscript and explain why they were used. Please note that such variables should not be used as proxies for other socially constructed/relevant variables (for example, race or ethnicity should not be used as a proxy for socioeconomic status).*<br>*Provide clear definitions of the relevant terms used, how they were provided (by the participants/respondents, the researchers, or third parties), and the method(s) used to classify people into the different categories (e.g. self-report, census or administrative data, social media data, etc.)*<br>*Please provide details about how you controlled for confounding variables in your analyses.*
Population characteristics	Human cartilage samples were collected from patients underwent total knee arthroplasty (TKA), and categorized into four groups, the Lean Lateral and the Lean Medial were obtained from individuals with lower BMI (<24, n=18). The Obesity Lateral and the Obesity Media were obtained from individuals with higher BMI (>28, n=22). The medical records of each patient werelisted in Supplementary Table 1.<br>Human synovial fluid samples were collected from patients diagnosed with OA and underwent arthrocentesis or arthroplasty. Patients underwent classification into three stages guided by the Kellgren & Lawrence Grade. The mild stage involves KL grades 0 and 1, indicating minimal OA or normal conditions (n = 40). The middle stage comprises KL grade 2, representing moderate OA(n = 36). The severe stage encompasses KL grades 3 and 4, signifying advanced or end-stage OA (n = 44). Furthermore, patients were classified based on their BMI levels. The "Lean Group" included individuals with a BMI < 24 (n = 52), whereas the "Obese Group" encompassed those with a BMI > 28 (n = 40).<br>All recruited patients did not have rheumatoid arthritis, which disease can affect the cartilage and synovial membrane. The OA patients with metabolic diseases including diabetes and hypertension were not excluded because of the research purposes to elucidate the regulatory mechanism between metabolic disorder and osteoarthritis.
Recruitment	Patients with a osteoarthritis undergoing arthroplasty or arthrocentesis were recruited for offering the cartilage tissue or synovial fluid. The Ethics Committee of Sir Run Run Shaw Hospital approved the use of these tissues,and written informed consent was obtained from all patients before the operative procedure. Recruitment bias is unlikely to impact this study as no patient comparisons are performed and no analysis of demographic or clinical covariates on cellular properties is performed.
Ethics oversight	This study was approved by the Ethics Committee of Sir Run Run Shaw Hospital (grant number SRRSH20230743)

Note that full information on the approval of the study protocol must also be provided in the manuscript.

Field-specific reporting

Please select the one below that is the best fit for your research. If you are not sure, read the appropriate sections before making your selection.

[x] Life sciences   [ ] Behavioural & social sciences   [ ] Ecological, evolutionary & environmental sciences

For a reference copy of the document with all sections, see nature.com/documents/nr-reporting-summary-flat.pdf

Life sciences study design

All studies must disclose on these points even when the disclosure is negative.

Sample size	While no statistical methods predetermined sample sizes for in vitro and in vivo analyses, preliminary experiments estimated assay variances to determine sufficient sample sizes, which are similar to those reported in other publications.
Data exclusions	No data were excluded from this study.
Replication	All experiments were performed independently at least three times. The numbers of biologically independent samples, mice per group, or human specimens are indicated in the figure legends. Data were presented as mean ± SD or SEM, or as minimum to maximum. Western blot pictures, confocal microscopy images, or histological images typically are from a representative experiment and the number of independent repeats is clearly indicated in the figure legends.
Randomization	For in vitro experiments, cultures were randomly assigned to different treatments. For mouse experiments, mutant and WT littermates were grouped based on sex, age, and genotype. Male mice were selected for the experiment in order to avoid concerns about hormonal effect in female mice. Mice were randomly allocated to high-fat diet, DMM surgery, or intra-articular injection groups without subjective judgment.
Blinding	Blinded individuals performed cartilage destruction analysis, synovial inflammation scoring, and immunohistochemical or immunofluorescence analysis, unaware of the specific mouse strains, treatment groups, or control versus experimental status.

Reporting for specific materials, systems and methods

We require information from authors about some types of materials, experimental systems and methods used in many studies. Here, indicate whether each material, system or method listed is relevant to your study. If you are not sure if a list item applies to your research, read the appropriate section before selecting a response.

Materials & experimental systems

n/a	Involved in the study	
☐	☒	Antibodies
☐	☒	Eukaryotic cell lines
☒	☐	Palaeontology and archaeology
☐	☒	Animals and other organisms
☒	☐	Clinical data
☒	☐	Dual use research of concern
☒	☐	Plants

Methods

n/a	Involved in the study	
☒	☐	ChIP-seq
☒	☐	Flow cytometry
☒	☐	MRI-based neuroimaging

Antibodies

Antibodies used

Western blotting:
HADHA (1:1000, Abcam, Cat#ab203114)
HADHB (1:2000,Abcam,Cat#ab230667)
SOX9 (1:2000, Abcam, Cat#ab185230)
MMP3 (1:2000, Abcam, Cat#ab52915)
MMP13(1:2000, Abcam, Cat#ab39012)
Acetyl Lysine (1:1000, Abcam, Cat#ab21623)
Ubiquitin (1:1000, Abcam, Cat#ab134953)
TSG101 (1:1000, Abcam, Cat#ab125011)
Phospho-ACC (Ser79) (1:1000, Cell Signaling Technology, Cat# 11818)
ACC (1:1000, Cell Signaling Technology, Cat# 3676)
Phospho-AMPKα (Thr172) (1:1000, Cell Signaling Technology, Cat# 2531)
AMPK (1:1000, Cell Signaling Technology, Cat# 5831)
Phospho-AMPK Substrate Motif (1:1000, Cell Signaling Technology, Cat# 5759)
CD63 (1:1000, Proteintech, Cat#67605-1-Ig)
TRIM9 (1:1000, Proteintech, Cat#10786-1-AP)
ACAN (1:1000, Millipore, Cat#C8035)
Histone H3K27ac (1:1000, Active Motif, Cat#39133)
COL2A1 (1:2000,Bioss, Cat#bs-10589R)
Acetyl-HADHA at Lysine 728 (1:1000, homemade, this paper)
FLAG HRP (1:10000, Sigma-Aldrich, Cat#A8592)
HA HRP (1:10000, GenScript, Cat#A01296)

Immunohistochemistry or immunofluorescence:
SOX9 (1:500, Abcam, Cat#ab185230)

HADHA (1:250, Abcam, Cat#ab203114)
HADHB (1:1000,Abcam,Cat#ab230667)
MMP13(1:500, Abcam, Cat#ab39012)
Phospho-ACC (Ser79) (1:500, Cell Signaling Technology, Cat# 11818)
COL2A1 (1:250,Bioss, Cat#bs-10589R)
ACAN (1:250, Millipore, Cat#C8035)
PDK4 (1:500, Proteintech, Cat#12949-1-AP)
Acetyl-HADHA at Lysine 728 (1:1000, homemade, this paper)

CUT&Tag assay:
Histone H3K27ac (1 μg per 50 μl reaction, Active Motif, Cat#39133)

Validation

All the following antibodies used in this study were validated by the suppliers:
HADHA (https://www.abcam.cn/products/primary-antibodies/hadha-antibody-epr17940-ab203114.html)
HADHB (https://www.abcam.cn/products/primary-antibodies/hadhb-antibody-ab230667.html)
SOX9 (https://www.abcam.cn/products/primary-antibodies/sox9-antibody-epr14335-ab185230.html)
MMP3 (https://www.abcam.cn/products/primary-antibodies/mmp3-antibody-ep1186y-ab52915.html)
MMP13 (https://www.abcam.cn/products/primary-antibodies/mmp13-antibody-ab39012.html)
Acetyl Lysine (https://www.abcam.cn/products/primary-antibodies/acetyl-lysine-antibody-chip-grade-ab21623.html)
Ubiquitin (https://www.abcam.cn/products/primary-antibodies/ubiquitin-antibody-epr8830-ab134953.html)
TSG101 (https://www.abcam.cn/products/primary-antibodies/tsg101-antibody-epr7130b-ab125011.html)
Phospho-ACC (Ser79) (https://www.cellsignal.com/products/primary-antibodies/phospho-acetyl-coa-carboxylase-ser79-d7d11-rabbit-mab/11818)
ACC (https://www.cellsignal.com/products/primary-antibodies/acetyl-coa-carboxylase-c83b10-rabbit-mab/3676)
Phospho-AMPKα (Thr172) (https://www.cellsignal.com/products/primary-antibodies/phospho-ampka-thr172-antibody/2531)
AMPK (https://www.cellsignal.com/products/primary-antibodies/ampka-d5a2-rabbit-mab/5831)
Phospho-AMPK Substrate Motif (https://www.cellsignal.com/products/primary-antibodies/phospho-ampk-substrate-motif-lxrxx-ps-pt-multimab-rabbit-mab-mix/5759)
CD63 (https://www.ptglab.com/Products/CD63-Antibody-67605-1-Ig.htm)
TRIM9 (https://www.ptglab.com/products/TRIM9-Antibody-10786-1-AP.htm)
ACAN (https://www.sigmaaldrich.com/GB/en/product/sigma/c8035)
Histone H3K27ac (https://www.activemotif.com/catalog/details/39133/histone-h3-acetyl-lys27-antibody-pab)
COL2A1 (https://www.biossusa.com/products/bs-10589r)
FLAG HRP (https://www.sigmaaldrich.com/GB/en/product/sigma/a8592)
HA HRP (https://www.genscript.com.cn/antibody/A01296-THE_HA_Tag_Antibody_HRP_mAb_Mouse.html)

Eukaryotic cell lines

Policy information about cell lines and Sex and Gender in Research

Cell line source(s)

Primary murine articular chondrocytes were extracted from the tibial plateaus of 7-day-old male mice (wild-type or Hadhafl/fl) through digestion with 0.5% collagenase II.
C28/I2 chondrocytes (Sigma-Aldrich, Cat#SCC043)
HEK293T cells (ATCC,Cat#CRL-11268)
3T3-L1 preadipocytes (ATCC, Cat#CL-173)

Authentication

None of these cell lines were authenticated by us.

Mycoplasma contamination

We confirmed that cell lines used were negative for mycoplasma contamination.

Commonly misidentified lines
(See ICLAC register)

No commonly misidentified cell lines were used.

Animals and other research organisms

Policy information about studies involving animals; ARRIVE guidelines recommended for reporting animal research, and Sex and Gender in Research

Laboratory animals

C57BL/6 mice were acquired from Hangzhou QiZhen Laboratory Animal Technology Center. ACAN-CreERT2 mice were kindly gifted by Prof. Ximei Wu (Zhejiang University School of Medicine). Col2-CreERT2 mice were obtained from the Jackson Laboratory. Hadhafl/fl, Hadhbfl/fl, and HadhaCKI/CKI (p. K728R Conditional Knock in) mice were purchased from Cyagen Biology Technology. For the generation of mice with Hadha, Hadhb conditionally knock-out, and Hadha (p. K728R) Conditional knock-in (CKI) in chondrocytes, Hadhafl/fl, Hadhbfl/fl, and HadhaCKI/CKI mice were bred with Acan-CreERT2 or Col2-CreERT2 mice. All mice were housed in a controlled environment with a temperature maintained at around 21°C, a 12-hour light/dark cycle, and ad libitum access to food and water.

Wild animals

This study did not involve wild animals.

Reporting on sex

Male mice were selected for the experiment in order to avoid concerns about hormonal effect in female mice.

Field-collected samples	This study did not involve animals collected from the field.
Ethics oversight	All animal experimental protocols were approved by the Institutional Animal Care and Use Committee of Zhejiang University (grant number ZJU20230529), in accordance with institutional guidelines.

Note that full information on the approval of the study protocol must also be provided in the manuscript.

Plants

Seed stocks	*Report on the source of all seed stocks or other plant material used. If applicable, state the seed stock centre and catalogue number. If plant specimens were collected from the field, describe the collection location, date and sampling procedures.*
Novel plant genotypes	*Describe the methods by which all novel plant genotypes were produced. This includes those generated by transgenic approaches, gene editing, chemical/radiation-based mutagenesis and hybridization. For transgenic lines, describe the transformation method, the number of independent lines analyzed and the generation upon which experiments were performed. For gene-edited lines, describe the editor used, the endogenous sequence targeted for editing, the targeting guide RNA sequence (if applicable) and how the editor was applied.*
Authentication	*Describe any authentication procedures for each seed stock used or novel genotype generated. Describe any experiments used to assess the effect of a mutation and, where applicable, how potential secondary effects (e.g. second site T-DNA insertions, mosiacism, off-target gene editing) were examined.*
